# B cell signatures and tertiary lymphoid structures contribute to outcome in head and neck squamous cell carcinoma

**DOI:** 10.1038/s41467-021-23355-x

**Published:** 2021-06-07

**Authors:** Ayana T. Ruffin, Anthony R. Cillo, Tracy Tabib, Angen Liu, Sayali Onkar, Sheryl R. Kunning, Caleb Lampenfeld, Huda I. Atiya, Irina Abecassis, Cornelius H. L. Kürten, Zengbiao Qi, Ryan Soose, Umamaheswar Duvvuri, Seungwon Kim, Steffi Oesterrich, Robert Lafyatis, Lan G. Coffman, Robert L. Ferris, Dario A. A. Vignali, Tullia C. Bruno

**Affiliations:** 1 Department of Immunology, University of Pittsburgh, Pittsburgh, PA USA; 2 Tumor Microenvironment Center, Hillman Cancer Center, University of Pittsburgh, Pittsburgh, PA USA; 3 Program in Microbiology and Immunology, University of Pittsburgh School of Medicine, Pittsburgh, PA USA; 4 Hillman Cancer Center, Pittsburgh, PA USA; 5 Department of Medicine, University of Pittsburgh, Pittsburgh, PA USA; 6 Department of Otolaryngology, University of Pittsburgh, Pittsburgh, PA USA; 7 Division of Hematology and Oncology, Department of Medicine, Hillman Cancer Center, Pittsburgh, PA USA; 8 Department of Otorhinolaryngology, University Duisburg-Essen, Essen, Germany; 9 Department of Pharmacology and Chemical Biology, University of Pittsburgh, Pittsburgh, PA USA; 10 Women’s Cancer Research Center, Magee-Womens Research Institute, University of Pittsburgh, Pittsburgh, PA USA; 11 Cancer Immunology and Immunotherapy Program, UPMC Hillman Cancer Center, Pittsburgh, PA USA

**Keywords:** Tumour immunology, Tumour immunology, Head and neck cancer

## Abstract

Current immunotherapy paradigms aim to reinvigorate CD8^+^ T cells, but the contribution of humoral immunity to antitumor immunity remains understudied. Here, we demonstrate that in head and neck squamous cell carcinoma (HNSCC) caused by human papillomavirus infection (HPV^+^), patients have transcriptional signatures of germinal center (GC) tumor infiltrating B cells (TIL-Bs) and spatial organization of immune cells consistent with tertiary lymphoid structures (TLS) with GCs, both of which correlate with favorable outcome. GC TIL-Bs in HPV^+^ HNSCC are characterized by distinct waves of gene expression consistent with dark zone, light zone and a transitional state of GC B cells. Semaphorin 4a expression is enhanced on GC TIL-Bs present in TLS of HPV^+^ HNSCC and during the differentiation of TIL-Bs. Our study suggests that therapeutics to enhance TIL-B responses in HNSCC should be prioritized in future studies to determine if they can complement current T cell mediated immunotherapies.

## Abstract (web summary)

Recent studies have highlighted the importance of B cells and tertiary lymphoid structures (TLS) in the modulation of anti-tumor immune responses. Here, the authors characterize how HPV status influences the phenotype of tumor infiltrating B cells in patients with head and neck squamous cell carcinoma and demonstrate that TLS with germinal centres are associated with better survival.

## Introduction

Immunotherapies targeting the programmed cell death protein 1 (PD1) pathway are approved by the Food and Drug Administration for the treatment of several metastatic or unresectable cancers including head and neck squamous cell carcinoma (HNSCC), but only ~20% of patients achieve a clinical benefit, highlighting the need for new therapeutic targets^1,2,3^. Tumor infiltrating B cells (TIL-Bs) represent a possible new target to compliment T cell-based immunotherapies, as they are frequent in many human tumors and positively correlate with favorable patient outcomes^4–7^. Specifically, increased TIL-Bs have been reported in cancers caused by environmental exposure to carcinogens (i.e., tobacco, alcohol, UV exposure) such as lung cancer and melanoma as well as cancers caused by viral infection such as hepatocellular carcinoma (HCC) and Merkel cell carcinoma (MCC)^6,8–11^. HNSCC offers a unique avenue to study TIL-Bs in the tumor microenvironment (TME) as HNSCC cancer can be caused by both exposure to environmental carcinogens or infection with high-risk human papillomavirus (HPV)^12^. Patients with HPV^+^ HNSCC have historically had better outcomes compared to HPV^–^ patients^13,14^. While the mechanisms underlying this difference in outcomes remains unknown, TIL-B are more frequent in HPV^+^ versus HPV^–^ HNSCC^5,15,16^. Understanding B cell phenotypes and the spatial organization of immune populations in the TME of patients in both viral and carcinogen-induced cancers will provide critical insight into how TIL-Bs can be leveraged to enhance antitumor immunity.

Tertiary lymphoid structures (TLSs) are immune aggregates with varying degrees of organization that form outside of secondary lymphoid organs (SLOs) in response to chronic inflammation or infection^17,18^. TLS are characterized by organization patterns similar to SLOs with defined T cell zones, B cell rich follicles and mature dendritic cells (DCs)^19,20^. TLSs have been shown to also correlate with increased patient survival in many human tumors^21,22^. Recent studies have demonstrated that the presence of B cells and TLS in melanoma, renal cell carcinoma, sarcoma, and HNSCC are associated with better responses to immune checkpoint blockade (ICB)^6,7,23,24^. However, TLSs are quite heterogeneous structures^25^, and the composition of TIL-Bs within these structures has not been fully elucidated. Characterization of TLS in the TME, including their composition, spatial organization, and maturity would provide critical insight into the roles these structures play in antitumor immunity. Additionally, understanding the factors that drive the formation of TLS would permit the identification of therapeutic avenues to foster an influx of antitumor TIL-Bs into the TME.

One feature associated with mature TLS is the formation and presence of germinal centers (GCs)^26^. GCs are typically found in SLOs and are responsible for producing affinity matured and class switched B cells that effectively recognize their cognate antigen, leading to memory B cells and durable humoral immunity. In humans, GC B cells are commonly identified as CD38^+^ IgD^−^ and transcription factor BCL6^+^. GC B cells can be further divided into centroblasts (dark zone; DZ) and centrocytes (light zone; LZ) through the expression of CXCR4 and CD86, respectively. In addition, recent studies have indicated Semaphorin 4A (SEMA4A) expression on human GC B cells in SLOs^27^. However, SEMA4A expression on GC TIL-B has not been previously reported in human cancer. Ultimately, GCs within TLS in the TME are indicative of maximal engagement of the humoral arm of the immune system in antitumor immune responses. In support of this, GC-like TIL-Bs were found to be increased in melanoma patients who responded to ICB^6^. Understanding the features that drive TIL-Bs toward a GC phenotype and contribute to the development and maintenance of GCs within TLS in the TME would provide a path to enhancing antitumor immunity in patients.

Here, we demonstrate that TIL-Bs in HPV^+^ and HPV^−^ HNSCC have distinct transcriptional signatures. GC TIL-Bs and TLS with GC are increased in HPV^+^ HNSCC patients and correlate with better outcomes. SEMA4A expression is increased on GC TIL-Bs compared to other TIL-B subsets and is associated with TIL-B differentiation and TLS containing GCs in HNSCC. GC TIL-Bs in HPV^+^ HNSCC are characterized by distinct waves of gene expression consistent with dark zone, light zone, and transitional state of GC B cells. Overall, this study demonstrates the importance of TIL-B transcriptional signatures, phenotypes and spatial patterning within the TME of patients with HNSCC, suggesting that this understudied lineage contributes to outcome and could be clinically targeted to increase antitumor immunity.

## Results

### Distinct TIL-B transcriptional signatures in HNSCC are revealed by scRNA-seq

**Fig. 1:**
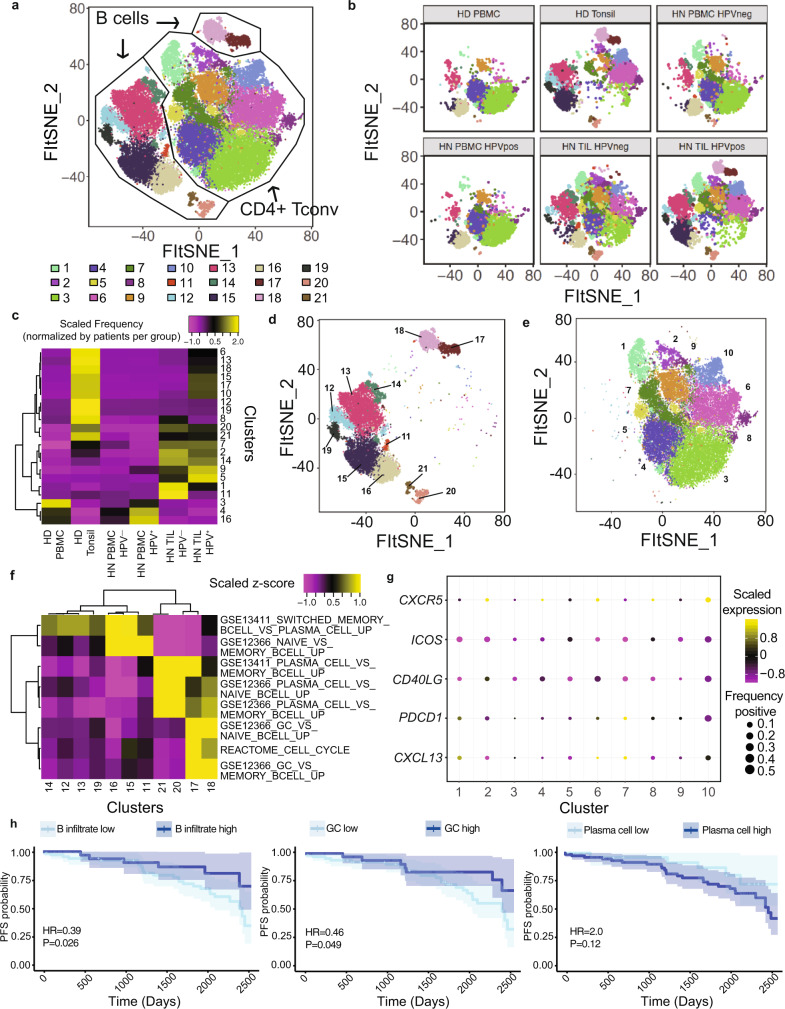
Differences in tumor-infiltrating B cell and helper CD4+ T cells between HPV− and HPV+ HNSCC contribute to survival. **a** Unsupervised clustering of 16,965 B cells and 30,092 helper CD4^+^ T cells (total of 47,057 cells) from all samples in patient cohort 1 (*n* = 6 healthy donor PBMC, *n* = 5 tonsils from sleep apnea patients, and paired blood and tumor specimens from *n* = 18 patients with HPV− disease and *n* = 9 patients with HPV+ disease). **b** Same FItSNE plot as **a** but showing clusters by sample type. **c** Heatmap showing the frequencies of cells recovered from each cluster by sample types, where the frequencies of cells were normalized by the number of patients assessed in each group. Tonsil samples, HPV− and HPV+ TIL were enriched for specific clusters. Statistical assessment of observed versus expected cell frequencies are detailed in Supplementary Fig. 3. **d**, **e** FItSNE plot (**d**) showing the clusters containing B cells from **a**, and the gene sets associated with specific functions for each cluster (**e**). Canonical B cell lineages, including naïve, switched memory, plasma cells and germinal center B cells were recovered. Interestingly, cells from HPV+ patients had GC B cells, while these cells were largely absent from TIL of HPV− patients. HPV− patients had a higher frequency of naïve and memory B cells. **f**, **g** FItSNE plot (**f**) showing the CD4^+^ helper T cells from **a**, and a dot plot highlighting the presence of cells with a T follicular-helper signature (cluster 10). **h** Progression-free survival (PFS) analysis derived from stratification of HNSCC TCGA patients based on enrichment scores for B cell infiltration, GC B cells, and plasma cells. Gene sets used to calculate enrichment scores were derived from our scRNAseq analysis and applied to bulk mRNAseq data from the TCGA (Methods). Cox proportional hazard models using a log-rank test were used for PFS analysis. The shaded regions represent 95% confidence intervals for the survival curves. Survival curves are derived from 111 HNSCC patients from the TCGA. Source data are available as a Source Data file.

We first analyzed scRNAseq data generated from purified CD45^+^ cells (i.e. all immune cells) from a total of 63 samples, including paired PBL and TIL from 18 patients with HPV^–^ HNSCC and 9 patients with HPV^+^ HNSCC (Supplementary Table 1, Cohort 1). We developed and validated a two-step approach to robustly identify B cells and CD4^+^ T_conv_ (Supplementary Fig. 1**;** Methods). We then bioinformatically isolated B and CD4^+^ T_conv_ and performed Louvain clustering (Methods) to reveal a total of 21 clusters (Fig. 1a). Next, we visualized the association between sample type and transcriptional signatures by interrogating the Fast interpolation-based t-distributed stochastic neighbor embedding (FItSNE) of cells from each sample type (Fig. 1b; “Methods”)^28^. Differential localization in the FItSNE revealed distinct transcriptional profiles associated with each sample type (Fig. 1b), and association between clusters and sample types (Fig.1c). Based on our cell type classifications (Supplementary Fig. 2), clusters 11 through 21 were B cells (Fig. 1d), while clusters 1 through 10 were CD4^+^ T_conv_ cells (Fig. 1e). To ascertain the role of B cells in each cluster, we filtered gene sets from the Molecular Signatures Data Base Immunologic Signatures (C7) to eight gene sets associated with canonical B cell function (Methods). This gene set enrichment analysis revealed B cell clusters associated with naïve (clusters 11, 15, 16), switched memory (clusters 12, 13, 14, 19), GC B cells (cluster 17 and 18) and plasma cells (clusters 20 and 21) (Fig. 1f). We observed statistically significant enrichment of GC TIL-Bs in the TME of HPV^+^ patients, while plasma cells were not statistically different in HPV^−^ versus HPV^+^ patients (Supplementary Fig. 3). We note that a subset of HPV^−^ patients had higher levels of plasma cells **(**Supplementary Fig. 3). Interestingly, GC TIL-Bs and GC B cells from healthy tonsils were overlapping, suggesting that there is little difference between GC signatures despite being within the TME versus SLOs. We also investigated CD4^+^ T_conv_ and identified a cluster that was strongly associated with a T_FH_ cell signature (i.e. high frequency and magnitude of *CXCR5, PDCD1, ICOS, CXCL13* expression; Fig. 1g). These data ultimately revealed increased GC TIL-Bs in HPV+ patients and increased plasma cells in HPV^−^ patients. Further, a T_FH_ signature was more pronounced in HPV+ disease.

To assess whether B cell signatures were clinically significant, we utilized bulk mRNAseq expression data available through The Cancer Genome Atlas (TCGA; Methods). Briefly, we scored each patient for the enrichment of B cell signatures derived from our data (Methods). Genes reflective of an overall B cell signature were derived from differentially expressed genes associated with B cells versus other major canonical immune lineages (Supplementary Data 1), while genes for GC B cells and plasma cells were derived from their respective clusters in the analysis of B cells and CD4^+^ T_conv_ alone (Supplementary Data 2). We then determined if these gene signatures were associated with progression free survival (PFS). Overall, high B cell infiltrate and high enrichment for GC B cells were positively associated with longer PFS (HR 0.39 to 0.46; *p* values of 0.026 and 0.049, respectively; Fig. 1h). Conversely, a high frequency of plasma cells trended toward shorter PFS (HR = 2.0, *p* = 0.12). We also found that enrichment scores for GC B cells from the light zone (LZ) were strongly correlated with those for T_FH_ cells, while there was no relationship between dark zone (DZ) and T_FH_ enrichment scores (Supplementary Fig. 4). Taken together, these data suggest that TIL-Bs in the HPV^+^ TME may be productively activated and receive CD4^+^ T_FH_ cell help.

Given the differences in transcriptional profiles between TIL-Bs from HPV^+^ and HPV^–^HNSCC, we performed bulk B cell receptor (BCR) sequencing via Adaptive (Supplementary Fig. 5; “Methods”). This analysis revealed no differences in measures of clonality or V-, D-, or J-gene usage between BCRs from HPV^−^ and HPV^+^ HNSCC. Thus whether TIL-Bs recognize tumor antigens in both types of HNSCC or viral antigen in HPV^+^ HNSCC will need to be further evaluated with more extensive cohorts or alternative assays in future studies.

### Germinal center TIL-Bs and TLSs are increased in HPV+HNSCC

**Fig. 2:**
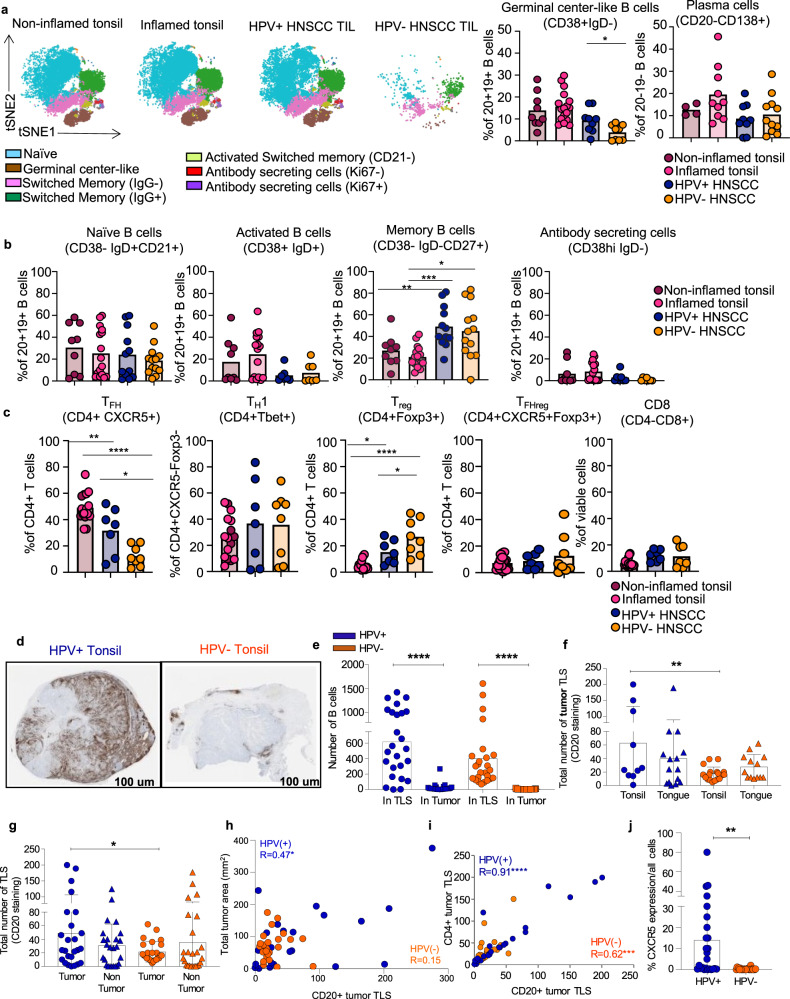
High-dimensional flow cytometry and immunohistochemistry reveal distinct TIL-B phenotypes and increased tertiary lymphoid structures in HPV+ HNSCC. **a** viSNE plots of B cells collected from non-inflamed and inflamed tonsils, HPV+ and HPV− HNSCC TIL and paired PBL (Supplementary Fig. 7) were analyzed using Cytobank. Non-inflamed tonsil (*n* = 4), inflamed tonsil (*n* = 6), HPV + HNSCC (*n* = 3), HPV− HNSCC (*n* = 2). Bar plot displaying frequencies of GC B cells and plasma cells in non-inflamed tonsil (*n* = 9), inflamed tonsil (*n* = 16), HPV+ HNSCC (*n* = 9), HPV− HNSCC (*n* = 9). *P = 0.02 Student‘s 2-sided *t* test. **b** Bar plot for frequency of B cell subpopulations. Non-inflamed tonsil (*n* = 9), inflamed tonsil (*n* = 16), HPV+ HNSCC (*n* = 12), HPV− HNSCC (*n* = 13). **P = 0.004, ***P = 0.0009, *P = 0.03, One way ANOVA followed by Tukey’s multiple comparisons test. **c** Frequencies of T follicular helper (T_FH_), regulatory T follicular helper (T_FHreg_), regulatory T cell (T_reg_), T helper type 1 (T_H_1) and CD8 T cells in non-inflamed tonsil (*n* = 6), inflamed tonsils (*n* = 10), HPV+ TIL (*n* = 7), HPV− TIL (*n* = 8). *P = 0.01,**P = 0.009,****P < 0.0001. *P = 0.04, *P = 0.03, ****P < 0.0001. One-way ANOVA followed by Tukey’s multiple comparisons test. **d** Representative CD20+ IHC on HPV+ and HPV− HNSCC tumors (×4 magnification). **e** B cell infiltrate counted within tumor bed compared to TLS. Total numbers from *n* = 50, 25 HPV+, 25 HPV− were graphed. ****P < 0.0001, Student‘s 2-sided *t* test. **f** Tumor TLS by site within the oropharynx (tonsil vs. tongue). Total numbers from *n* = 50, 25 HPV+, 25 HPV− were graphed. **P = 0.0096, Student‘s 2-sided. Data are presented as mean values ± SEM. **g** Total number of tumor TLS and non-tumor TLS numbers in HPV+ and HPV− disease. Total numbers from *n* = 50, 25 HPV+, 25 HPV− were graphed. *P = 0.0249, Student‘s 2-sided *t* test. Data are presented as mean values ± SEM. **h** Correlation of CD20^+^ tumor TLS with tumor area. Total tumor area (mm^2^) for each patient tumor was calculated by a pathologist. *P < 0.05, non-parametric Spearman correlation. **i** Total tumor TLS independently counted for CD20^+^ and CD4^+^ (*n* = 50, 25 HPV+, 25 HPV−). ****P < 0.0001, ***P < 0.001, non-parametric Spearman correlation. **j** Total CXCR5 was scored for all cell types (*n* = 50, 25 HPV+, 25 HPV−).**P = 0.0012, Student‘s 2 sided *t* test. Source data are provided as a Source Data file.

As transcriptional analysis revealed differential enrichment of TIL-Bs in HPV^+^ and HPV^–^ HNSCC, we developed a spectral cytometry panel (Methods) to validate our scRNAseq findings at the protein level and to determine if there were any additional alterations in TIL-B subpopulations in HNSCC. We first quantified frequencies of TIL-Bs and plasma cells in HNSCC primary tumors (Supplementary Table 2, Cohort 2), which revealed a significant increase in CD19^+^CD20^+^ TIL-Bs compared to plasma cells in the TME (Supplementary Fig. 6a,b). Next, we utilized our spectral cytometry panel to perform unsupervised clustering of B cells on a subset of HNSCC patients (**Cohort 2**). In these patients, we identified seven distinct B cell clusters: naïve B cells (CD38^−^IgD^+^CD27^−^), IgG^+^ switched memory B cells (IgG^+^CD38^−^IgD^‒^CD27^+^), IgG^−^ switched memory B cells (IgG^−^CD38^−^IgD^−^CD27^+^), activated switched memory (CD38^−^IgD^−^CD27+CD21^−^), GC B cells (CD38^+^IgD^−^BCL6^+^SEMA4a^+^), tissue-like memory B cells (CD38^−^IgD^−^IgG^+^CD27^−^CD21^−^FcRL5^+^) and antibody-secreting cells (CD38^hi^CD27^+^Ki67^+/−^) (Fig. 2a, Supplementary Table 2, Cohort 2). Tonsil and HPV+ HNSCC tumors were enriched for naïve, switched memory, and GC B cell clusters while HPV− tumors were enriched for switched memory clusters (Fig. 2a). Of note, overall TIL-B density is increased in HPV^+^ TIL compared to HPV^−^ TIL (Fig. 2a). HPV^+^ and HPV^−^ PBL were enriched for naïve and switched memory B cell clusters (Supplementary Fig. 7a). Interestingly, we observed that the tissue-like memory B cells were only present in the HNSCC PBL (Supplementary Fig. 7a, b). To quantify differences in TIL-B subsets identified in our unsupervised clustering, we used standard flow cytometry gating and pooled data from additional HNSCC patients within Cohort 2 that were stained with a modified flow cytometry panel (“Methods”). GC TIL-B was significantly increased in HPV^+^ HNSCC compared to HPV^−^ HNSCC (Fig. 2a). We did not observe a significant difference in plasma cell frequency between HPV^+^ and HPV^−^ HNSCC patients (Fig. 2a). We also did not observe a significant difference in naïve, activated or antibody-secreting B cells in HPV^+^ and HPV^−^ HNSCC (Fig. 2b). Of note, total class-switched memory B cells (CD38^−^IgD^−^) are significantly increased in both HPV^+^ and HPV^−^ HNSCC when compared to normal and inflamed tonsils (Fig. 2b).

As our transcriptional analysis of CD4^+^ T cells in HNSCC tumors revealed an increased CD4^+^ T_FH_ cell signature in HPV^+^ HNSCC, we sought to interrogate the frequencies of CD4^+^ T_conv_ lineages (i.e. T_FH_, T_H1_, regulatory T_FH_, and T_regs_) present in HNSCC patients by flow cytometry. We observed a significant increase in T_FH_ within HPV^+^ HNSCC patients compared to HPV^−^ patients (Fig. 2c), but T_H1_ cells were not significantly different. Regulatory T_FH_ (CXCR5^+^ FOXP3^+^) was not significantly different between HPV^+^ and HPV^−^ tumors (Fig. 2c). T_reg_ were significantly increased in HPV^–^ HNSCC patients compared to HPV^+^ HNSCC and normal and inflamed tonsils, and CD8^+^ T cell frequencies were comparable (Fig. 2c).

Although frequencies of cells quantified by flow cytometry are informative, evaluating spatial localization of cells in situ within the TME is an orthogonal approach that contextualizes the TME in which immune cells are located. We utilized a separate cohort (Supplementary Table 3, Cohort 3) with significant patient follow up for these locational studies. We first used single-plex immunohistochemistry (IHC) to evaluate the number and location of TIL-Bs within different areas of the oropharynx. We observed that B cells predominantly infiltrated TLS regardless of HPV status and that TLS formation was dictated by HPV status regardless of tissue sites i.e. tonsil vs. tongue (Fig. 2d–f). Next, we evaluated frequencies of TLS in the tumor versus outside the tumor in HPV^–^ and HPV^+^ HNSCC (Fig. 2g). HPV^+^ tumors had a higher frequency of TLS within and adjacent to the tumor and the HPV+ tumors had a significant correlation with the total tumor area whereas HPV^−^ tumors did not (Fig. 2h). Further, the number of CD4^+^ T cells and TIL-Bs in TLS were strongly correlated (Fig. 2i). Finally, we found a higher frequency of CXCR5^+^ immune cells (consistent with a T_FH_ CD4^+^ T_conv_ infiltrate) in HPV^+^ TIL versus HPV^−^ TIL (Fig. 2j), confirming that TLS likely foster GC reactions in the TME. Taken together, these flow cytometric and spatial data confirm that GC B cells and CD4^+^ T_FH_ are present within TLS and are more frequently found in HPV^+^ HNSCC patients.

### SEMA4A expression is associated with GC B cell differentiation and TLS with GC in HNSCC

**Fig. 3:**
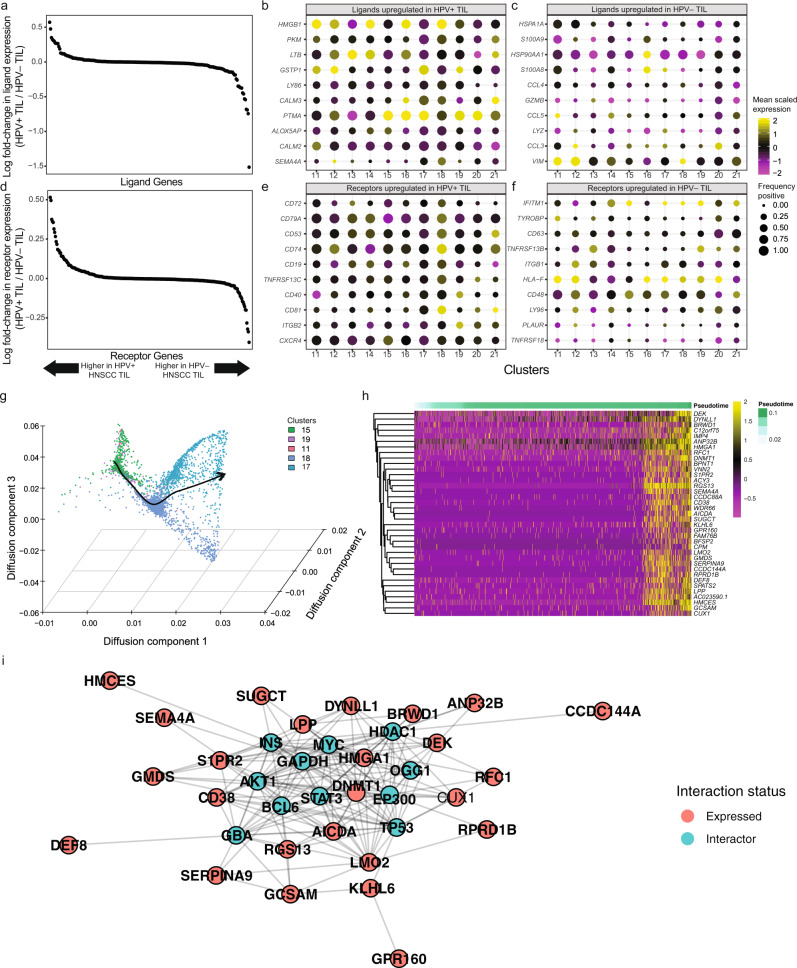
Differentially expressed ligands and receptors in HNSCC and modeling of GC differentiation identify *SEMA4A* as associated with development and maturation of GC. **a** Differential expression of ligands by B cells in the TME of HPV– and HPV+ HNSCC. Number of patient samples is the same as Fig. 1. **b** Number of cells expressing ligands and magnitude of expression in HPV+ TIL-B by cluster. Consistent with GC B cell and formation of TLS, *LTB* was one of the top expressed ligands across HPV+ TIL-B. *SEMA4A* expression was largely restricted to clusters 17 and 18, which are GC TIL-B. **c** Expression of top ligands by HPV− TIL-B included several chemokines (*CCL4* and *CCL5*). **d** Differential expression of receptors by B cells in the TME of HPV– and HPV+ HNSCC. **e** Top receptors expressed by HPV+ TIL-B including genes associated with GC function including *CD40* and *CXCR4*. **f** Top receptors in HPV– B cells included *CD63*, which is associated with downregulation of CXCR4 and is suppressed by Bcl6. **g** Diffusion map embedding of B cell associated with a lineage spanning naïve and GC B cells identified by slingshot (Methods). B cells are shown by their clusters identified in Fig. 1, and the line connecting the clusters denotes the differentiation trajectory with increasing pseudotime. **h** Heatmap showing the top genes that are temporally associated with CD38 expression dynamics during progression from naïve B cells to GC B cells. *SEMA4A* follows the same expression dynamics as *CD38*. A total of 1000 cells were randomly sampled from the entire naïve cell to GC B cell dataset for visualization in the heatmap. **i** Inferred protein-protein interactions from Cytoscape for the top genes that share temporal dynamics with *CD38*. Nodes represent genes, and edges in the network represent putative protein-protein interactions. BCL6, p53, AKT, and MYC were all inferred to be interacting with proteins encoded by genes that follow the expression dynamics of *CD38*. All analysis in this figure is derived from all patients in our scRNAseq cohort.

To better understand differences between TIL-B in HPV^+^ versus HPV^–^ HNSCC, we next utilized our scRNAseq data to interrogate the expression of ligands and receptors in the TME (Cohort 1). We found several ligands in the TME associated with each type of HNSCC (Fig. 3a) and visualized the top 10 in each type of HNSCC (Fig. 3b, c). Interestingly, we found that *SEMA4A* was enriched in HPV+ HNSCC and was largely restricted to GC B cell clusters (i.e. clusters 17 and 18), relative to other clusters. We performed a similar analysis with receptors, and found several receptors associated with GC B cells in HPV^+^ TIL (e.g. *CD40* and *CXCR4*), and others associated with plasma cells in HPV^–^ TIL (e.g. *CD63* and *LY96*) (Fig. 3d–f).

We next used pseudotemporal modeling to better elucidate the dynamics of gene expression as cells progress from naïve B cells to GC B cells. These analyses are important not only to trace differentiation to GC B cells, but also organization of B cells into TLS, as naïve B cells must be pulled into a GC reaction to create a functional GC. Pseudotemporal modeling can be used to reconstruct differentiation trajectories from scRNAseq data based on smooth changes in gene expression that take place across cells as they transition from one state to the next. We found a trajectory from naïve to GC B cells (Fig. 3g), which allowed us to infer a pseudotime ordering of B cells during differentiation from naïve to GC B cells. Interestingly, this analysis revealed that *SEMA4A* is associated with the transition from naïve to GC B cells and shares similar dynamics of expression with *CD38* (Fig. 3h). We also tracked the dynamics of genes associated with CD38 in order to infer putative protein–protein interactions using Cytoscape (Methods) that may be taking place as B cells differentiate from naïve to GC B cells (Fig. 3h). This analysis revealed extensive interactions, including interactions with *BCL6*, the master transcriptional regulator associated with GC B cells (Fig. 3i). Functional enrichment revealed a variety of pathways associated with this interaction network, including Epstein-Barr infection as a top hit as well as several pathways associated with metabolic changes that occurring during progression to GC G cells (Supplementary Data 3). Taken together, this analysis revealed that *SEMA4A* expression is enriched in GC TIL-Bs, and the temporal expression of *SEMA4A* is associated with differentiation into GC TIL-Bs.

**Fig. 4:**
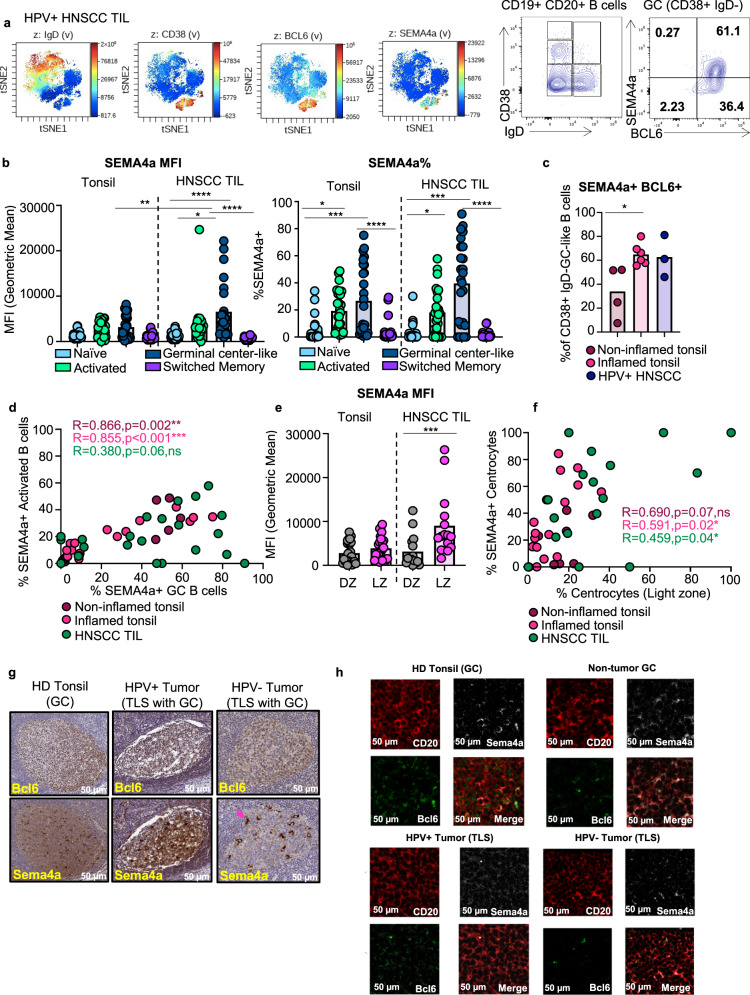
SEMA4a expression is increased in GC TIL-Bs in TLS in HNSCC. **a** Individual viSNE feature plots demonstrating expression level of canonical markers used to identify B cell subpopulations in Fig. 2a. Number of samples is the same as Fig. 1. Representative flow plot showing traditional flow gating strategy for B cell subsets quantified in Fig. 2a, b and SEMA4a co-expression with BCL6. CD19+CD20+ B cells were gated on CD38 and IgD. CD38+ IgD− (GC B cells) were than gated on SEMA4a and BCL6 **b** Bar plot showing geometric mean fluorescence intensity (gMFI) of SEMA4A on B cell subsets. Statistical analysis by ordinary one-way ANOVA followed by Tukey’s multiple comparisons test. *P = 0.02 **P = 0.0013, ***P = 0.0007, ***P = 0.0004, ****P < 0.0001 Bar plot showing frequencies of SEMA4A positivity on B cell subsets. **c** Bar plot comparing the frequency of SEMA4a^+^ BCL6^+^ GC B cells. Statistical analysis by ordinary one-way ANOVA followed by Tukey’s multiple comparisons *P = 0.02. **d** Scatter plot comparing the frequency of SEMA4a+ GC-B cells to SEMA4a^+^ activated B cells. Statistical analysis by Spearman correlation. **P = 0.002 ***P < 0.001 ns; not significant **e** Bar plot showing MFI of SEMA4a on dark zone (DZ) and light zone (LZ) GC B cells. Statistical analysis by Student‘s two-sided *t* test (Mann–Whitney) **P = 0.001. **f** Scatter plot comparing the frequency of SEMA4a^+^ light zone GC-B cells to total light zone GC B cells. Statistical analysis by Spearman correlation. *P = 0.02, *P = 0.04, ns; not significant **g** Representative IHC for BCL6 and SEMA4A in HNSCC patients. BCL6 and Sema4a expression was compared in HPV+ and HPV− HNSCC patients to HD tonsil. Pink arrow is pathological characterization of macrophage. **h** Confocal imaging of TLS in HPV+ and HPV− tumors compared to GC in HD tonsil. CD20, SEMA4A, and BCL6 were co-localized to visualize co-expression of SEMA4A and BCL6 which mark GC in HD tonsil. Scale bar is 50 µm for all images. Source data are provided as a Source Data file.

We next sought to interrogate whether SEMA4A has a similar expression pattern at the protein level on TIL-B (Cohort 2). Indeed, SEMA4A was co-expressed with CD38 as in the transcriptomic data (Fig. 4a). Additionally, SEMA4A was co-expressed with BCL6, a key transcription factor that regulates germinal centers in SLOs (Fig. 4a, c). We also found that SEMA4A mean fluorescence intensity (MFI) and frequency was significantly increased on GC TIL-Bs compared to GC and activated B cells in healthy donor tonsil via our high dimensional flow cytometry (Fig. 4a, b). In addition, SEMA4A MFI and frequency were significantly increased on GC TIL-Bs compared to memory or naïve TIL-Bs in HNSCC tumors (Fig. 4b). Lastly, we observed an increase in costimulatory molecules such as CD40 and CD86 on activated TIL-Bs compared to naïve TIL-Bs in HNSCC tumors (Fig. 4a and Supplementary Fig. 7c), which we expect to be upregulated on B cell populations like GC and activated B cells for optimal antigen presentation. Pseudotemporal ordering in our scRNAseq data suggested that *SEMA4A* expression is increased during differentiation towards GC, meaning *SEMA4A* may play a role in the progression of activated B cells. To interrogate this, we assessed whether there was a correlation between SEMA4A^+^ activated B cells and SEMA4A^+^ GC B cells and found a significant positive correlation between the two groups in healthy and inflamed tonsil. There was a trend towards a positive correlation between SEMA4A^+^ activated TIL-B cells and SEMA4A^+^GC TIL-Bs that did not reach statistical significance (Fig. 4d). Overall, these data suggest that SEMA4A may play a role in the development and maturation of B cells into GC B cells.

B cells entering the GC reaction begin in the dark zone (DZ) where they undergo expansion and somatic hypermutation^29,30^. Centroblasts then follow a CXCL13 gradient to enter the light zone (LZ), where they capture antigen presented on follicular dendritic cells (FDCs) which they present to CD4^+^ T_FH_ cells in order to undergo selection^30^. Since we observed significantly less GC TIL-Bs in HPV^–^ HNSCC tumors, we sought to determine if there were any additional aberrations in SEMA4A expression on GC TIL-B cell subsets in HNSCC tumors. Specifically, we assessed expression on DZ or LZ GC TIL-Bs. SEMA4A was significantly expressed on LZ GC TIL-B cells in HNSCC tumors (Fig. 4e). Further, SEMA4A^+^ LZ GC TIL-Bs positively correlate with the frequency of total LZ GC TIL-Bs (Fig. 4f). This suggests SEMA4A could be important in both the development of GC B cells and the interactions between LZ GC B cells and T_FH_ cells in normal and tumor tissues. Using IHC, we confirmed the presence of SEMA4A and co-expression of the canonical GC transcription factor BCL6 with SEMA4A in tonsils (Fig. 4g). Interestingly, while SEMA4A is on B cells and myeloid cells in healthy donor (HD) tonsils and HPV^+^ tumors, it is more restricted to macrophages (pink arrow) in HPV^−^ tumors. To compliment the single-plex IHC studies, we also performed 3 color confocal microscopy to interrogate co-localization of Sema4a and Bcl6 within TLS in HPV^+^ and HPV^−^ HNSCC tumors (Fig. 4h). Within HD tonsil GC and non-tumor GC (GC within tumor-adjacent normal oropharyngeal tissue), SEMA4A and BCL6 co-localize as expected. Co-localization of these two markers and BCL6 expression in general is increased within TLS in HPV+ patients compared to TLS in HPV− patients. Taken together, these data demonstrate that SEMA4A is associated with both activated and GC B cells in tonsil and the TME of patients with HNSCC, ultimately marking TLS with GC in HPV^+^ patients due to its strong correlation with BCL6 expression. While SEMA4A may govern the formation of GCs within TLS in HNSCC patients, further interrogation of this pathway is necessary to understand its role in TLS formation and maturity in the TME.

### Dissection of germinal center B cell reactions reveals distinct waves of gene expression

**Fig. 5:**
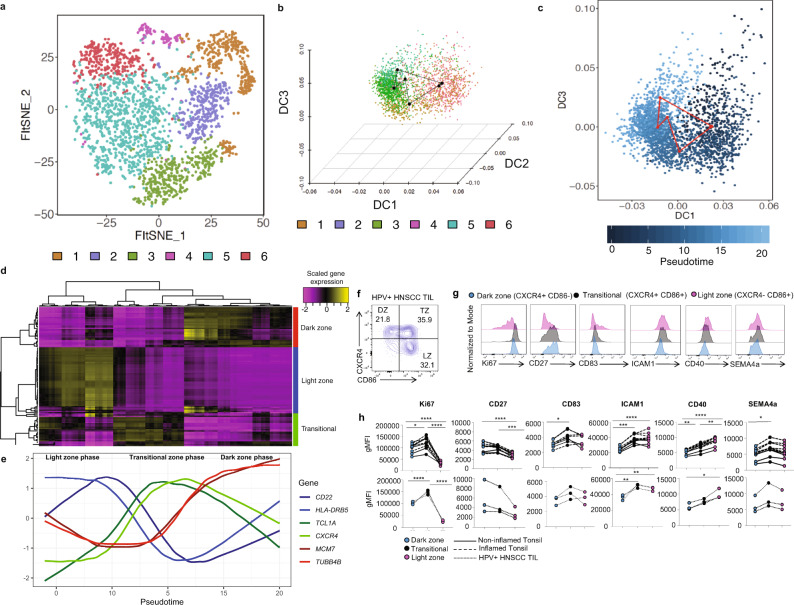
Cyclical pseudotime modeling of germinal center B cell reactions reveals waves of gene expression. **a** FItSNE showing 6 clusters of germinal center B cells (i.e. cells from clusters 17 and 18 from Fig. 1A/D). **b** Three-dimensional diffusion map embedding of germinal center B cells, with cells colored by their cluster identities from **a**. Black dots represent the centroid of each cluster, and the lines connecting the black dots represent the circular path through germinal center reactions. **c** DCs 1 and 3 captured most of the information required to reconstruct the circular trajectory of germinal center B cells (left panel). Pseudotemporal ordering was inferred by fitting the equivalent of a non-parametric principal component from the center of the trajectory using the assumption that the data is on a closed curve (right panel). **d** Loess regression was used to fit curves for the top 20 differentially expressed genes across GC B cell clusters as a function of pseudotime inferred in **c**. Genes were found to cluster into three distinct groups by fit with pseudotime. Clusters were defined as dark zone, light zone, and transitional. Analysis in **a**–**d** is derived from six healthy blood donors, five tonsils, and 27 HNSCC patients. **e** Marker genes derived from **d**, with scaled gene expression plotted as a function of time. Blue genes correspond to light zone (LZ) GC B cells, green genes correspond to B cells moving between LZ and dark zone (DZ) GC B cells, and red genes correspond to DZ GC B cells. **f** Representative flow plot showing gating strategy for LZ, DZ, and TZ populations. CD38+ IgD^−^ BCL6^+^ (GC B cells) were then gated on CXCR4 and CD86. Dark zone (DZ) CXCR4^+^ CD86^−^, “Transitional” (TZ) (CXCR4^+^CD86^+^) and Light zone (LZ) (CXCR4^−^CD86^+^) were identified. **g** Representative plots comparing expression of the three distinct GC TIL-B populations, DZ (blue-filled histogram), TZ (black-filled histogram) LZ (pink-filled histogram) **h** Scatter plots quantifying differences in geometric mean fluorescence intensity of indicated key markers on the three distinct GC B cell populations in tonsils and HPV+ HNSCC. Statistical analysis by ordinary one-way ANOVA followed by Tukey’s multiple comparisons test. *P = 0.02, **P = 0.002, ***P = 0.0002, ****P = < 0.0001. Source data are provided as a Source Data file.

Since a better understanding of GC reactions has implications for antitumor immunity and effective humoral immunity in infection and vaccination, we performed an in-depth transcriptional dissection of GC reactions. To achieve this, we first bioinformatically isolated GC B cells and re-clustered them to reveal more subtle differences within the canonical GC populations (Fig. 5a). This analysis revealed 6 clusters with distinct gene expression patterns (Fig. 5a). Typical pseudotime algorithms assume a linear differentiation trajectory, but with GC B cells we expect a cyclical process as B cells toggle between LZ and DZ interactions for optimal B cell maturation. Thus, we developed a computational approach (see “Methods”) to capture the cyclical nature of this process. First, we embedded cells in a diffusion space, yielding a cyclical topology (Fig. 5b and Methods). We then connected each cluster via their centroids and fit a principal curve to infer a pseudotime score for each cell in the GC (Fig. 5c). We then evaluated genes associated with GC progression, and identified not only DZ and LZ reactions, but also a transitional state for TIL-Bs within our cyclical GC model (Fig. 5d). When viewed as a function of pseudotime, we found three distinct waves of expression associated with each of these GC states within the cyclical process (Fig. 5e). The first phase consisted of expression of canonical LZ genes such as *CD22* and *HLA-DRB1*, followed by a wave of transitional genes consisting of *CXCR4* and *TCL1A*, followed by a final wave of cell cycle genes which are consistent with the proliferative nature of DZ B cells. The code utilized for this computational approach is publicly available in an R package called “circletime”^31^.

Using the top differentially expressed genes of each GC cell state (DZ, LZ, and transitional [TZ]) we sought to validate these GC cell states in normal SLO tissue and HNSCC via flow cytometry. The key genes that were upregulated on DZ were CXCR4, CD27, CD72, CCR1. We identified the upregulation of CD40, CD37, CD7, and CD180 on LZ as well as CD83, ICAM1, CCR6, and CD18 on TZ. Using the classical gating strategy for human GC B cells (CD38^+^ IgD^−^ BCL6^+^), we first assessed CXCR4 and CD86 expression as these canonically define DZ and LZ GC B cells (Fig. 5f). We observed DZ GC B cells (CXCR4^+^ CD86^−^), TZ, (CXCR4^+^ CD86^−^), and LZ (CXCR4^−^ CD86^+^) in HPV^+^ HNSCC and normal and inflamed tonsils (Fig. 5f, Supplementary Fig. 8a, b). While we observed protein expression of CD37, CD7, CD72, CCR6, CD18, and CD180 on GC B cells in tonsils and HPV^+^ HNSCC, we observed no significant difference in expression of these markers between the three distinct GC cell states (Supplementary Fig. 7b). GC B cells in the DZ undergo rapid proliferation, thus we next looked at Ki67 expression in each GC B cell population. Surprisingly, we observed significantly higher Ki67 expression in the TZ GC B cells compared to DZ or LZ in both tonsils and HPV^+^ HNSCC (Fig. 5h, i). These data suggest that TZ GC B cells may be the population undergoing proliferation in GCs in human tonsils and HPV^+^ HNSCC tumors. CD83 is also used to distinguish DZ and LZ in human and mice with expression being predominantly on LZ GC B cells^32,33^. Recently, a cell state for GC B cells termed “gray zone (GZ)” that co-expresses CXCR4 and CD83 was identified using RNA-sequencing and flow cytometry in mice^33^. We assessed CD83 expression on DZ, TZ, LZ and found significantly higher expression on TZ compared to DZ in tonsils (Fig. 5g, h). Given the expression of CXCR4, Ki67, and CD83 on TZ in human tonsils and HPV^+^ HNSCC, future studies should assess whether TZ GC B cells in humans are analogous to GZ GC B cells in mice. CD27 expression was previously shown to be higher on DZ GC B cells in human tonsil^32^. Indeed, we observed significantly higher CD27 expression on DZ compared to LZ in tonsils and observed a similar trend in HPV^+^ HNSCC but it was not significant (Fig. 5h).

Next, we compared ICAM1 and CD40, as these may be important for T_FH_ interactions in the LZ. We observed significantly higher expression of ICAM1 and CD40 on LZ compared to DZ in both tonsils and HPV^+^ HNSCC tumors, however, ICAM1 was not significantly different between LZ and TZ (Fig. 5g, h). CD40 expression was significantly higher on the LZ compared to the TZ in tonsils but not in HPV^+^ HNSCC (Fig. 5g, h). Our initial assessment of SEMA4A expression revealed that LZ GC B cells expressed significantly higher SEMA4A expression compared to DZ in HNSCC tumors (Fig. 4e). Interestingly, we found increased SEMA4A expression on TZ GC B cells compared to DZ in tonsils but not significantly different between TZ and LZ (Fig. 5g, h). We found a similar trend in HPV^+^ HNSCC, although it did not reach statistical significance (Fig. 5g, h). A complete understanding of the transitional state of GC B cells in humans will contribute to the identification of the signals that lead to egress from GC reactions, factors that contribute to the cycling between DZ and LZ, and key cues that are necessary for a bonified GC reaction in the TME.

### TLSs are associated with better survival in HNSCC

**Fig. 6:**
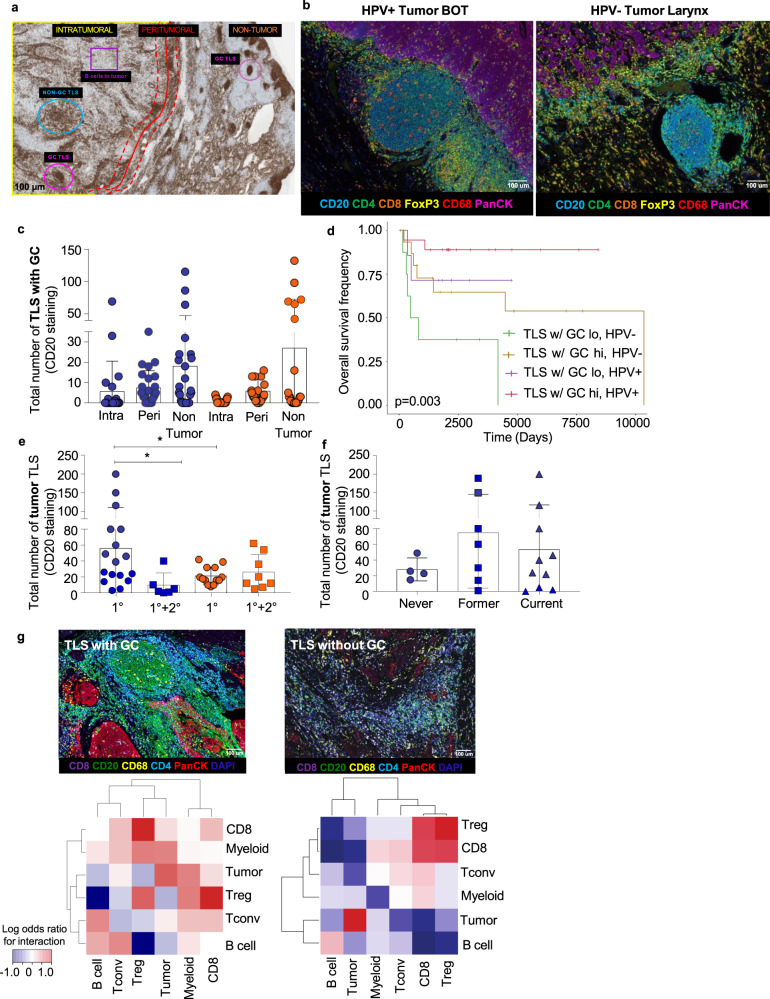
Increased TLS with GC within HPV+ HNSCC patients correlate with increased patient survival. **a** Annotated tumor section stained for CD20 via single-plex IHC from a HNSCC patient (×20 magnification). Annotations for tumor (intratumoral and peritumoral) and non-tumor areas are indicated. Blue circle =TLS without GC, Pink circle = TLS with GC, Purple square = TIL-B infiltrate within tumor bed. **b** Representative Vectra staining for TLS with GC within HPV+ and HPV− HNSCC tumors. BOT = base of tongue. **c** TLS with GC are increased intratumorally (intra) and peritumorally (peri) in HPV+ HNSCC patients. Differences in intra vs. peri TLS with GC trended toward an increase in HPV+ HNSCC patients. Data are presented as mean values ± SEM. **d** TLS with GC in the tumors of HPV+ and HPV− HNSCC patients correlate with increased patient survival. Cox proportional hazard was used to evaluate overall survival based on high versus low frequencies of TLS with GC and HPV status (*p* = 0.003, log rank test). The hazard ratio for high versus low TLS with GC was 0.32, and the hazard ratio for HPV+ versus HPV− was 0.27. **e** Total number of tumor TLS are increased in HPV+ patients that do not progress to secondary disease. Total tumor TLS (via CD20^+^ staining) were compared by primary disease (1°) vs. primary and secondary disease (1°+2°). *n* = 50, 25 HPV+, 25 HPV−.*P = 0.0336 and 0.0281, Student’s 2 sided *t* test. Data are presented as mean values ± SEM. **f** Total number of tumor TLS are increased in former and current smokers that are also HPV+. Total tumor TLS (via CD20^+^ staining) were compared in HPV+ patients that were never smokers vs. former or current smokers. Data are presented as mean values ± SEM. **g** Cell–cell neighborhoods in TLS with GC are distinct compared to TLS without GC (“Methods”). Top panels show a TLS with GC (left) and a TLS without GC (right). Bottom panels show the odds ratio of proximity to other cell types (Methods), with red representing a high probability of interaction with a given cell type and blue a low probability of interaction. Source data are provided as a Source Data file.

To complement the transcriptional analysis of GC reactions in HNSCC tumors, we evaluated the number of TLS with GC in HNSCC tumors, as GCs are paramount for maximal B cell immunity^30^. In counting TLS with GCs outside the tumor, we found elevated TLS with GC in HPV^+^ and HPV^−^ tumors (Fig. 6a, b, Supplementary Table 3, Cohort 3). However, these TLS with GCs were increased intratumorally and peritumorally in HPV^+^ patients (Fig. 6c). Of note, an intratumoral increase in TLS with GCs in HPV^+^ HNSCC supports previous studies in other virally induced human tumors^34^. Furthermore, TLS with GC in the tumor correlated with increased survival in both HPV^+^ and HPV^–^ disease (Fig. 6d), but more discretely in HPV^+^ disease, most likely due to better overall survival in these patients^13^. We also performed multivariate survival analysis including TLS with GC, HPV status, and disease burden (as measured by the number of positive nodes; Supplementary Fig. 9). In addition, we revealed that HPV^+^ HNSCC patients with increased disease burden (i.e. primary and secondary disease) had significantly less tumor TLS in their primary disease compared to those individuals with primary disease alone (Fig. 6e). This suggests that tumor TLS could potentially be important for reducing tumor recurrence at the same site of the primary tumor (secondary disease). We also found that former and current smokers with the HPV^+^ cohort of patients had increased TLS compared to never smokers (Fig. 6f). This indicates the importance of other environmental cues in TLS formation in cancer. Finally, we analyzed the key cell-cell neighborhoods associated with TLS with GC vs. TLS without GC in HNSCC (Fig. 6g). In TLS with GC, TIL-Bs interact with other TIL-Bs and CD4^+^ T_conv_ TIL, which is in line with the working definition of an active GC. Interestingly, an evaluation of TLS without GC in HNSCC revealed that TIL-Bs were not frequently in the same neighborhood with CD4^+^ T_conv_, and instead CD8^+^ TIL and T_regs_ were implicated as a dominant interaction. These results demonstrate that in TLS with GC, the spatial patterning becomes distinct from well-infiltrated tumors where immune cells are found in aggregates.

## Discussion

In this study, we sought to perform an in-depth analysis of B cells in the TME of patients with HNSCC, with the goal of improving our understanding of the immunobiology of B cells and the potential role they have in generating baseline antitumor immune responses. Our study integrated new technical approaches across three cohorts of patient samples (*n* = 124) and suggests that not only higher numbers of TIL-Bs, but also the specific phenotype and localization of TIL-Bs in the TME contribute to overall survival. Interestingly, we have implicated SEMA4A^+^ GC TIL-Bs and TLS with GC as increased in HPV^+^ HNSCC patients compared to HPV^−^. Further, we also identified CD4^+^ T_FH_ in the TME of HNSCC, which complements findings in breast and colorectal cancer^35–37^. The correlation we observed between LZ B Cells and T_FH_ in the TME extends this finding further, demonstrating the importance of crosstalk between CD4^+^ T cells and GC TIL-Bs and the need for CD4^+^ T cell help for GC TIL-B survival in the TME of HNSCC. Our single-cell transcriptional characterization of TIL-B populations uncovered numerous states of B cells in the TME and revealed distinct differences between HPV^+^ and HPV^−^ HNSCC. These differences should be considered in the development of a B cell-focused immunotherapy for HNSCC.

B cells are a heterogenous population with phenotypically and functionally distinct subsets. Thus, characterization of TIL-B phenotypes in treatment naïve patients is a critical first step in the development of B cell-focused immunotherapies. However, B cell targeted therapies may need to enhance certain subsets of B cells while inhibiting others, necessitating more dissection of the change in TIL-B phenotypes following therapy. For example, in melanoma, patients who did not respond to standard of care immunotherapy i.e. anti-PD1 and/or anti-CTLA4 had significantly more naïve B cells than responders^6^. In this context, our findings suggest that driving naïve TIL-Bs towards activated and GC phenotypes could be one way to complement current immunotherapeutic strategies.

Functional assessment of TIL-B subpopulations is also needed to better inform potential targeting strategies. TIL-Bs in HNSCC have the potential to contribute to antitumor immunity in a number of ways including presenting tumor antigen to CD4^+^ T cells^38,39^. In NSCLC, TIL-Bs were shown to present tumor antigen to and influence the phenotype of CD4+ T cells in vitro^40^. A T_H_1 CD4^+^ TIL phenotype following antigen presentation correlated with activated TIL-Bs in NSCLC patients while “exhausted” TIL-Bs correlated with T_regs_^40^. We hypothesize that GC TIL-B in HPV^+^ HNSCC may be more equipped to present antigen to CD4^+^ T cells given their presence in TLS with GC and expression of CD40, CD86, and ICAM1, which are key proteins involved in interactions with T_FH_ cells. Additionally, TIL-Bs in HNSCC could potentially enhance antitumor immunity through the production of tumor-specific antibodies that can activate NK-mediated antibody-dependent cellular cytotoxicity (ADCC)^41–43^. Memory B cells, which are largely responsible for responding to secondary stimuli and differentiating into antibody-secreting cells are increased in both HPV^+^ and HPV^−^ HNSCC. Future studies in HNSCC should assess whether memory TIL-Bs can respond to antigen stimulation and differentiate into antibody-secreting cells. Given the suppressive TME of HPV^−^ HNSCC and a significant increase in T_reg_, it is possible memory TIL-B function could be impaired or suppressed. However, if memory TIL-B produces antibodies in HNSCC patients, antigen specificity of those antibodies should be addressed. It is unclear whether antibodies produced in HPV^+^ or HPV^−^ HNSCC are tumor-reactive or self-reactive. However, antibodies in the serum of HPV^+^ HNSCC patients have been shown to have specificity to early and late antigens of HPV16^43^. Additionally, recent studies have shown that antibodies produce by TIL-Bs within the TME of HPV^+^ HNSCC have specificity to HPV viral antigens E2, E6, and E7^44^. Whether these HPV-specific antibodies play a role in the anti-tumor response remains to be determined.

One function for TIL-Bs that is definitively correlated with increased survival and immunotherapeutic response in cancer patients is their role in TLS^11,19,20,23,45^. TLS formation and maintenance in tumors is an active area of investigation. Early studies reveal that common mechanisms of lymphoid organogenesis such as the presence of inflammatory cytokines and interactions of immune cells with tissue-resident stromal cells such as fibroblasts and mesenchymal stem cells are important for TLS initiation^19–21,46,47^. Our study identifies a potential mechanism for TLS formation in tumors through the identification of SEMA4a expression on GC TIL-Bs within TLS. *SEMA4A* is a membrane bound glycoprotein that is important for T cell co-stimulation and an important driver of T_H_2 responses in humans, and was recently found to be expressed on human GC B cells in SLOs^27^. Further, SEMA4a can interact with non-immune receptor Plexin D1 which is expressed on endothelial cells and immune receptor T cell, Ig domain, mucin domain-2 (Tim-2) and neuropilin-1 (NRP1) expressed by T cells^48–52^. Thus, SEMA4A may play a central role in generating immune aggregates via TIL-B interactions with endothelial and T cells. In fact, CD4^+^ T_FH_ expresses high levels of NRP1^51^. Future studies should more thoroughly characterize the factors that lead to the creation of effective TLS, or conversely the factors that inhibit TLS formation in the TME, especially because TLS are both predictive^45,53,54^ of and correlated with response to immunotherapy^6,7,23^. There are a multitude of ways in which B cells can contribute to antitumor immunity, and it will be important to link B cell subsets with specific antitumor function to inform therapeutic strategies.

Current immunotherapeutic regimens aim to reinvigorate exhausted CD8^+^ TIL within the TME^55^. Overall, our findings suggest that the presence of GC TIL-Bs within TLS in treatment naive patients is associated with better outcomes. Focusing on driving TIL-Bs into TLS with GC is a potentially paradigm-shifting step towards new immunotherapies. For example, we found that SEMA4A may be a better marker of both early-stage and functional TLS in the TME compared with the canonical GC B cell marker BCL6. As such, determining ways to drive SEMA4A expression on TIL-B and determining which ligands are required to nucleate TLS is an obvious next step for B cell mediated immunotherapy development. These findings are likely to stimulate future studies involving SEMA4A in other cancers that have reported GC TIL-Bs such as lung cancer and melanoma^6,11,26^. In addition, the formation of TLS with GC both peritumorally and intratumorally is paramount for increased patient survival and are increased in HPV^+^ HNSCC. Thus, our study provides a rationale to assess SEMA4A expression on GC TIL-Bs and in TLS of other virally induced cancers such as HCC, MCC, and cervical cancer where SEMA4A expression on GC TIL-Bs has not yet been reported. Future studies should seek to evaluate how viral infection impacts the development and maintenance of GC TIL-B and TLS with GC in virally induced cancers. Further, additional environmental factors (i.e. the microbiome of the oral cavity and oropharynx) should be queried in future studies. Lastly, improved analysis of spatial relationships will be paramount as our data suggest that GC biology within TLS is associated with favorable antitumor immunity. Beyond cancer, our dissection of B cell biology can inform strategies aimed at enhancing vaccine responses, or conversely disrupting the generation of B-cell mediated immune activation to suppress autoimmunity. Ultimately, this study highlights the significance of phenotypes and spatial patterns of TIL-Bs in both virally and carcinogen-induced HNSCC and suggests that future studies should investigate if therapeutic enhancement of humoral immunity in HNSCC can complement current immunotherapeutic strategies.

## Methods

### Patient cohorts

Several patient cohorts were used for various aspects of this manuscript. Figures 1, 3, 5 and Supplementary Figs 1–4 used the patient cohort^56^ described in Supplementary Table 1. This cohort consisted of consecutive patients undergoing surgical resection as treatment for head and neck cancer at the University of Pittsburgh, patients undergoing tonsillectomy as treatment for sleep apnea or tonsilitis, or healthy donors. Figures 2, 4 and Supplementary Figs 5–7 used the patient cohort described in Supplementary Table 2, and also consisted of patients undergoing surgical resection as treatment for head and neck cancer or patients undergoing tonsillectomy as treatment for sleep apnea or tonsilitis, or healthy donors. Figure 6 used the patient cohort described in Supplementary Table 3, and consisted of a retrospective cohort of patients with formalin-fixed paraffin-embedded samples. All patients provided informed written consent prior to donating samples for this study, and the study was approved by the Institutional Review Board (University of Pittsburgh Cancer Institute, Protocol 99-069).

### Blood and tissue processing

Peripheral blood was obtained by venipuncture and collected into tubes containing EDTA coagulant. Blood was processed into PBMC by Ficoll-Hypaque density gradient centrifugation. Briefly, whole blood was diluted and layered over Ficoll-Hypaque, followed by centrifugation at 400*g* for 20 min with the brake set to off. PBMC were then collected and washed in complete RPMI (i.e. RPMI 10% fetal bovine serum and 1% penicillin/streptomycin).

Tissues were collected from either HNSCC patients undergoing resection as treatment or sleep apnea or tonsillitis patient undergoing tonsillectomy. Tissues were collected directly into collection media (i.e. complete RPMI + 1% amphotericin B) in the operating room and were processed as soon as possible following surgery. For transcriptional analysis, samples were processed within 2 h of collection. Sample processing consistent of manually dissociating tumor tissue into approximately 1 mm pieces, then washing with cRPMI and passing the suspension over a 100 uM filter. The filter was then washed with cRPMI, and the cells were centrifuged at 500*g* for 5 min. If significant numbers of red blood cells were present, red blood cell lysis was performed as per the manufacturer’s instructions (BD Pharm Lyse).

### Flow cytometry-based cell sorting

For experiments requiring cell sorting, cells were first stained in PBS with 2% FBS and 1 mM EDTA for 15 min, followed by centrifugation at 500*g* for 5 min and staining with viability factor in PBS for 15 min. Cells were then centrifuged again, resuspended in PBS with 2% FBS and 1 mM EDTA, and sorted using a MoFlo Astrios High Speed Sorter (Beckman Coulter). Sort cells were collected directly in cRPMI. For single-cell RNAseq analysis, live CD45+ cells were sorted by using Fixable Viability Dye eFluor780 (eBioscience) and CD45 conjugated to PE (Biolegend, clone HI30).

### Single-cell RNAseq library preparation and sequencing

Immediately the following sorting, cells were centrifuged for 5 min at 500*g* and were resuspended in PBS with 0.04% BSA. Cells were then counted using the Cellometer Auto2000 (Nexcelom) and loaded into the 10X Controller (10X Genomics) targeting a recovery of 2000 cells per sample. Following bead/cell emulsification, RNA was reverse transcribed into cDNA. cDNA and was then purified by SPRI-bead selection and amplified, followed by fragmentation for library generated followed by 12 cycles of PCR amplification. The library quality was determined by Bioanalyzer analysis and concentration by KAPA qPCR DNA Quantification. Libraries were then pooled and sequenced on a NextSeq500 (University of Pittsburgh Genomics Research Core) using a high-output kit.

### Processing and clustering of single-cell RNAseq data

Following sequencing, raw Illumina reads were demultiplexed based on i7 indices (10X Genomics) using the mkfastq command of the CellRanger suite of tools (10X Genomics). Demultiplexed FASTQs were then aligned to the human genome (GRCh38) using the count command of CellRanger to generate cell/barcode matrices. Cell/barcode matrices were then read into Seurat (v2.3.4) for downstream analysis.

Clustering was performed as an initial analysis step for several scRNAseq datasets using the workflow implemented in Seurat. Briefly, raw reads were normalized for library size per cell and log transformed. Highly variable genes were identified and selected, followed by scaling and center of data as well as regression out technical variables (i.e. number of genes per cell, percent of reads aligning to ribosomal genes per cell and percent of reads aligning to mitochondrial reads per cell). These scaled and centered expression values were then used as input into a principal component analysis to reduce the dimensionality of the data. The top principal components that explained the most variance in the dataset were heuristically selected as input for the fast interpolation-based t-SNE^28^ and the Louvain-based clustering algorithm implemented in Seurat.

### Identification of cell types in single-cell RNAseq

We initially sorted and sequenced all cells of the hematopoietic lineage (i.e. CD45+ cells), and were therefore needed to robustly identify B cells and CD4^+^ T_conv_ for downstream in-depth analysis. We did this using a two-step semi-supervised identification strategy. This strategy consisted of first identifying core transcriptional programs of the major lineages of the immune compartment. To do this, we downloaded publicly available single-cell RNAseq data of sorted immune lineages (10X Genomics; https://www.10xgenomics.com/resources/datasets/). We then clustered these cell populations as described above to identify lineage-specific clusters. Once these clusters were identified, we performed differential gene expression analysis using a Wilcoxon rank sum test to identify the top 20 genes associated with each cluster. These genes were defined as the core transcriptional profile of each lineage. We then used these genes as gene sets to test individual cells for enrichment of each immune lineage. Briefly, we used the log-fold-change in gene expression as a metric and input these fold-changes into the Wilcoxon rank sum test for genes in each core lineage set versus genes outside that set, deriving a gene set score and p-value for each gene set for each cell. The core lineage gene set associated with the lowest p-value for each cell was then applied as that cell type. Following this test for each cell, we then examined clusters of cells in aggregate, and identified each cluster by the most common cell type enriched within that cluster. We then compared this two-step method (i.e. single-cell gene set enrichment testing and identification followed by aggregate identification of clusters) to the ground truth for each of the clusters know to be a sorted cell lineage using a confusion table from the R package caret. B cells and CD4+ T_conv_ were identified from all hematopoietic cells in our dataset using this two-step method.

### Quantification of differences in cell frequencies across clusters

We evaluated the enrichment of cells from a given sample type in each cluster by dividing the frequency of observed cells over expected cells in each cluster. The expected frequency of cells was calculated by assuming cells from each sample group were evenly divided across clusters. Analysis of variance was used to determine if the cell enrichment across groups was statistically significant, and Wilcoxon rank sum tests were used to determine if there were statistically significant differences in cell frequencies between HPV− and HPV+ TIL.

### Gene set enrichment analysis of B cells

Gene set enrichment analysis was performed using the R package singleseqgset as previously described^57^. Briefly, log-fold-change in gene expression was calculated for all genes across B cell clusters and used as input for a variance inflation corrected Wilcoxon rank sum test to whether sets of gene were upregulated in a concerted manner within a cluster. B cell gene sets were curated based on biological relevance from the Molecular Signatures Database (C7 Immunology Gene Sets).

### Survival analysis using The Cancer Genome Atlas

To determine if our gene sets were relevant for survival, we utilized bulk RNAseq data for HNSCC patients available through the TCGA and create an enrichment score for each signature from each patient as previously described^57^. Briefly, we derived genes sets that were reflective of the cell populations of interested and determined an enrichment score for each patient in the TCGA. Enrichment scores were calculated by using a Kolmogorov-Smirnov test comparing genes within the gene set of interest versus genes outside the given gene set. The gene set for B cell infiltration was defined by taking the top genes that were differentially expressed with a log fold-change >0.5 from the overall clustering used to define the major immune lineages. Gene sets for GC and plasma cells were derived by taking the top 200 differentially expressed genes by log fold-change from the two GC clusters and the two plasma cell clusters versus all B cells and CD4+ T cells (i.e. from cluster 17 and 18 for GC cells and 20 and 21 for plasma cells from Fig. 1a). We then stratified patients based on high versus low enrichment scores and performed Cox proportional hazards regression (see statistical analysis below).

### Pseudotime analysis of B cells

Clustering analysis is useful for grouping cell types based on similar gene expression patterns but does not capture information related to developmental trajectories of cells. To assess developmental trajectories, we first embedded cells in a low-dimensional diffusion map (e.g. performed non-linear dimensionality reduction^58^. We then used the R package slingshot^57^ to infer a pseudotime for each cell along the developmental trajectory, and to infer individual trajectories. To evaluate whether genes were statistically associated with pseudotime, we performed LOESS regression using the R package gam, where we fit gene expression as a function of pseudotime along each trajectory. We focused on the trajectory that was characterized by progression from naïve B cells to germinal center B cells.

For pseudotime analysis of germinal center B cells, slingshot could not be used since it assumes a linear trajectory. Germinal center B cells are in a cycle between light and dark zones, and therefore require pseudotime inference based on a cyclical process. Therefore, a principal curve was fit along the circular trajectory to infer the pseudotime of each cell in this process. Genes were once again investigated for their relationship to pseudotime and were clustered based correlation of gene expression over pseudotime. An R package called “circletime” and accompanying vignette were created to encapsulate the code necessary to generate all aspects of the cyclical pseudotemporal analysis.

### Adaptive B cell receptor sequencing

Adaptive Biotechnologies’ immunoSEQ platform was used to perform a survey of B cell receptors (BCRs) from HNSCC patients. Total DNA was isolated from cryopreserved snap frozen tumor tissues using the QIAGEN DNeasy Blood and Tissue Kit and was used as input for the immunoSEQ platform. Analysis was performed using Adaptive’s analysis interface. BCR sequencing data are available as Supplementary Data 4.

### Surface and intracellular antibody staining of patient and healthy donor cells

Single-cell suspensions from either HNSCC tissue, tonsillar tissue, HNSCC PBL or healthy donor PBL were stained with fluorescently labeled antibodies at 1:100 dilution (see Supplementary Table 4 for antibody panel information), for 25 mins at 4 °C in PBS (Thermo Fisher) supplemented with 2% FBS (Atlanta Biologicals) and 0.01% azide (Thermo Fisher) (FACS buffer). Cells were then washed with FACS buffer and spun down (1500 rpm, 5 min, RT). Cells were next stained using Fixable Viability Dye (eBioscience) in PBS to exclude dead cells. Cells were then washed with PBS and spun down (1500 rpm, 5 min, RT). For intracellular transcription factor staining cells were fixed using fixation/permeabilization buffer (eBioscience) for 20 mins at 4 °C the washed with permeabilization buffer (eBioscience). Cells were then stained with fluorescently labeled antibodies. Flow cytometry measurements were performed on an LSR-II flow cytometer (BD) using BD FACS Diva software or Cytek Aurora using SpectroFlo software (Cytek). All data were analyzed using FlowJo.

### High dimensional spectral cytometry

viSNE and FlowSOM analyses were performed on Cytobank (https://cytobank.org). FSC files for each sample type were first down sampled and concatenated in flowjo whereby each patients’ FSC file contributed an equal number of total events to the concatenated file. Files were uploaded into Cytobank and traditional flow cytometry gating was performed to gate live CD19 + CD20 + B cells. viSNE analysis was performed on CD19+ CD20+ B cells using proportional sampling cells from each concatenated FCS file to equal 100,000 total events with 1000 iterations, a perplexity of 30, and a theta of 0.5. The following markers were used to generate the viSNE maps: IgG, CD27, CD21, FcRL5, CCR1, Ki67, BAFFR, CD38, CD7, CD40, CD37, CCR6, CD72, IgD, ICAM1, CD180, CD72, CD86, CXCR4, SEMA4a, CD83, CD18. The resulting viSNE maps were fed into the FlowSOM clustering algorithm. A new self-organizing map (SOM) was generated using hierarchical consensus clustering on the tSNE axes. The SOM contained 100 clusters and 10 metaclusters for B cells were identified.

### Single-plex immunohistochemistry

Fresh tissues were formalin-fixed immediately followed surgical resection and were then embedded in paraffin. Tissues were processed as previously described^57^. Briefly, fixed tissues were then slide mounted, deparaffinized using xylene and ethanol, and then re-fixed in formalin for 15 min followed by antigen retrieval. Slides were stained with the following antibodies: CD20 (Clone L26, ThermoFisher, 1:100, Cat# MA5-13141), CD4 (Clone D7D27, Cell Signaling, 1:100, Cat# 25229), CXCR5 (Clone D6L36, Cell signaling, 1:100, Cat# 72172), Tbet (Clone 4B10, Abcam, 1:100, Cat# ab91109). Quantification of cells and TLS were performed by a HNSCC pathologist. Specifics of these quantifications are outlined in Fig. Legends and definitions of a TLS were consistent across three independent pathologists.

### Cytoscape analysis

We utilized Cytospace version 3.8.0 in conjunction with the top genes that shared temporal dynamics with *CD38* expression during differentiation from naïve B cells to GC B cells. The confidence score for interaction was set to 0.2, and the maximum number of additional interactors was set to 10. After pruning nodes that did not have any edges, we performed functional enrichment to identify biological functions associated with the interactions present in our network.

### Immunofluorescence analysis

Fresh tissues were formalin-fixed immediately followed surgical resection and were then embedded in paraffin. Formalin-fixed paraffin-embedded tissue sections were cut at 5um thickness and mounted on slides. Briefly, sections were baked at 60 °C for 2 h or overnight and deparaffinized using xylene and ethanol, followed by fixation in 10% neutral buffered formalin for 15 min. Tissues were then subjected to heat-induced epitope retrieval (HIER) cycles in AR9 or AR6 citrate buffers (Akoya Biosciences). Post antigen retrieval, slides were blocked for 10 min with blocking buffer, followed by incubation with primary antibodies for 30 min in a humidified chamber at room temperature. Secondary antibodies conjugated to horseradish peroxidase were then added for 10 min. Separate opal detector fluorophores were used for each marker as follows: CD4 (Clone RM, BioCare Medical, Prediluted, Cat# API3209 AA)/Opal540, CD8 (Clone C8/144B, BioCare Medical, 1:200, Cat# ACI31160A)/Opal570, CD20 (Clone L26, Leica Biosystems, 1:200, Cat# CD20-L26-L-CE)/Opal520, CD68 (Clone D4B9C, Cell Signaling, 1:800, Cat# 76437S)/Opal650, FOXP3 (Clone D608R, Cell Signaling, 1:250, Cat# 12653 S)/Opal620 and Pan-cytokeratin (Clone AE1/AE3, Santa Cruz Biotech, 1:100, Cat# SC81714)/Opal690. Opal 7 color manual kit (including all opal fluorophores and DAPI) was purchased from Akoya Biosciences (Cat# NEL811001KT, 1:600 dilution). Final round of antigen retrieval was carried out to counterstain cells with spectral DAPI. Stained tissue sections were then mounted and sealed with Diamond Anti-fade mounting media (Thermo Fisher, cat # P36970). Following staining, slides were imaged as whole slide scans on the Vectra (Perkin Elmer). Regions of interest were selected from the whole slide scans, and slides were re-imaged to captures these regions at 10x magnification. Images were unmixed after scanning using inForm and Phenochart. Using FIJI, cell segmentation was performed with watershed analysis in each individual channel, and cells were assigned an x- and y-position on each slide associated with a given channel and assigned a cell type based on the channel. We then performed Delaunay triangulation to determine to odds of a cell interaction with another given cell type based on proximity^57,59^.

### Immunofluorescent confocal microscopy

Formalin-fixed paraffin-embedded (FFPE) 4 µm slides were deparaffinized, rehydrated, and processed for heat-induced antigen retrieval. Samples were then washed with PBS and blocked against nonspecific binding using universal blocking buffer for 1 h at room temperature. Conjugated antibodies CD20/PE-cy7 (Clone 2H7, Biolegend, 1:50, Cat# 302312), BCL6/AF488 (Clone K112-91, BD Biosciences, 1:50, Cat# 561524) and SEMA4A/APC (Clone 5E3, Biolegend, 1:50, Cat# 148406) were diluted in 10% universal blocking buffer (5 µg/ml) and applied for 1 h at room temperature. Samples were then washed with PBS and mounted with antifade media and left to dry overnight at 4 °C. All images were acquired on Nikon A1 confocal microscope and analyzed using Nikon elements NIS.

### Statistical analysis

Analysis of variance (ANOVA) followed by pairwise *t* tests was used to compare more than two groups of continuous variables. Two groups of continuous variables were compared by t-tests or Wilcoxon rank sum tests were indicated. Tukey’s multiple comparisons test was performed following ANOVA where indicated. Survival analysis was performed by using Cox proportional hazards regression analysis, using either nominal values or stratifying continuous variables into nominal values. Stratification of continuous variables was performed using the “cutp” function of the R package survMisc. Correlations were performed using either Pearson’s correlation or Spearman’s correlation, as indicated. Correction for multiple comparisons using the false discovery rate was performed where appropriate. *P* values and false discovery rates were considered statistically significant when the two-sided type I error was 5% or less.

## Supplementary information

Supplementary Information

Descriptions of Additional Supplementary Files

Supplementary Data 1

Supplementary Data 2

Supplementary Data 3

Supplementary Data 4

## Data Availability

Unprocessed FASTQ files for scRNAseq data are available through the Sequence Read Archive SRP226817. Processed feature barcode matrices for all scRNAseq data are available through the Gene Expression Omnibus with accession number GSE139324. The bulk RNAseq and clinical data utilized for survival analysis from TCGA is available through the Broad Genome Data Analysis Center Firehouse (https://gdac.broadinstitute.org/). The gene barcode expression matrices from sorted cells are available through 10X Genomics website (https://www.10xgenomics.com/resources/datasets/). The Adaptive BCR sequencing data have been deposited in the ImmuneACCESS database (Adaptive Biotechnologies) under DOI: 10.21417/ATR2021NC and URL https://clients.adaptivebiotech.com/pub/ruffin-2021-nc. Source data are provided with this paper, and are available as a Source Data file. The remaining data are available within the Article, Supplementary Information or available from the authors upon request.

## Source data

Source Data

## Code availability

For analysis, standard workflows from the R packages Seurat and Destiny were utilized for normalization, dimensionality reduction, clustering, and diffusion maps. We developed a new R package called circletime for the analysis of cyclical pseudotemporal processes (www.github.com/arc85/circletime; DOI: 10.5281/zenodo.4599815)^31^.
