# Supplementary material for: B cell signatures and tertiary lymphoid structures contribute to outcome in head and neck squamous cell carcinoma
Source: Nat Commun. 2021 Jun 7;12:3349. doi: 10.1038/s41467-021-23355-x (PMC8184766; doi:10.1038/s41467-021-23355-x)
Supplement: Supplementary file 1 — Supplementary Information [file 41467_2021_23355_MOESM1_ESM.pdf]

	Healthy PBMCs	Healthy Tonsils	HNSCC
	N=6	N=5	N=27
**Demographics**			
Median Age	55(29-56)	38(28-53)	60(15-80)
Sex N (%) Female	3(50%)	0(0%)	6(22.2%)
**Tumor p16 Status**			
p16+ N (%)	N/A	N/A	9(33.3%)
p16- N (%)	N/A	N/A	18(66.7%
NE N (%)	N/A	N/A	0(0%)
**Site of primary tumor**			
Tonsil N(%)	N/A	N/A	3(11.1%)
Tongue N(%)	N/A	N/A	8 (29.6%)
Base of Tongue N(%)	N/A	N/A	4(14.8%)
Larynx N(%)	N/A	N/A	3(11.1%)
Other N(%)	N/A	N/A	9(33.3%)
**Pathalogical Staging**	N/A	N/A	
TX N(%)	N/A	N/A	1(3.7%)
T0 N(%)	N/A	N/A	0(0%)
T1-T2 N(%)	N/A	N/A	10(37%)
T3-T4A. N(%)	N/A	N/A	15 (55.6%)
Unknown N(%)	N/A	N/A	1 (3.7%)
**Pathalogical Node**			
NX N(%)	N/A	N/A	0 (0%)
N0 N(%)	N/A	N/A	8(29.6%)
N1-N2 N(%)	N/A	N/A	6(22.2%)
N2A-N2C N(%)	N/A	N/A	10(37.0%)
N3-N3B N(%)	N/A	N/A	2(7.4%)
Unknown N(%)	N/A	N/A	1(3.7%)
**Tobacco Use**			
Yes N(%)	0(0%)	1(20%)	14(52%)
No N(%)	4(66.7%)	1(20%)	8(29.6%)
Former N(%)	2(33.3%)	3(60%)	4(14.8%)
**ETOH use**			
Never N(%)	0(0%)	0(0%)	0(0%)
Yes N(%)	2(33.3%)	0(40%)	9(33.3%)
No N(%)	1(16.7)	2(40%)	11(25.6%)
Occasional N(%)	3(50%)	1(20%)	4(14.8%)
Former N(%)	0(0%)	0(0%)	0(0%)
Unknown N(%)	0(0%)	2(40%)	3(11.1%)

Supplementary Table 1: Clinical characteristics of prospective patient cohort for single-cell RNAseq and immunofluorescence (Cohort 1)

	Tonsils	HNSCC
	N=24	N=43
**Demographics**		
Median Age	31(18-52)	57 (36-81)
Sex N (%) Female	13(54.2%)	13(30.2%)
**Tumor p16 Status**		
p16+ N (%)	N/A	12(28%)
p16- N (%)	N/A	8 (18.6%)
NE N (%)	N/A	23(53.4%)
**Site of primary tumor**		
Tonsil N(%)	N/A	7(16.3%)
Tongue N(%)	N/A	8(18.6%)
Base of Tongue N(%)	N/A	6(13.9%)
Larynx N(%)	N/A	7(16.3%)
Mouth (N%)	N/A	6(13.9%)
Other (N%)	N/A	9(20.9%)
**Pathalogical Staging**		
TX N(%)	N/A	11(25.6%)
T0 N(%)	NA	1(2.33%)
T1-T2 N(%)	NA	10(23.3%)
T3-T4A N(%)	NA	21(48.8%)
**Pathalogical Node**		
NX N(%)	N/A	12(27.9%)
N0 N(%)	N/A	11(25.6%)
N1-N2 N(%)	N/A	10(23.2%)
N2A-N2C N(%)	N/A	4(9.3%)
N3-N3B N(%)	N/A	6 (14%)
**Tobacco Use**		
Yes N(%)	6(25%)	22(51.2%)
No N(%)	13(54.2%)	7(16.3%)
Former N(%)	5(20.8%)	14(32.5%)
**ETOH use**		
Never N(%)	1(4.2%)	0(0%)
Yes N(%)	13(54.2%)	28(65.1%)
No N(%)	8(33.3%)	7(16.3%)
Occasional N(%)	0(0%)	8(18.6%)
Former N(%)	2(8.3%)	0(0%)

Supplementary Table 2: Clinical characteristics of prospective patient cohort for spectral flow cytometry and protein validation (Cohort 2)

	HNSCC
	N=50
**Demographics**	
Median Age	60(37-82)
Sex N (%) Female	6(12%)
**Tumor p16 Status**	
p16+ N (%)	25(50%)
p16- N (%)	25(50%)
NE N (%)	0(0%)
**Site of primary tumor**	
Tonsil N(%)	25(50%)
Tongue N(%)	4(8%)
Base of Tongue N(%)	21(42%)
Larynx N(%)	0(0%)
Other N(%)	0(0%)
**Pathalogical Staging**	
TX N(%)	0(0%)
T0 N(%)	0(0%)
T1-T2 N(%)	34(68%)
T3-T4 N(%)	12(24%)
Unknown N(%)	4(8%)
**Pathalogical Node**	
NX N(%)	0(0%)
N0 N(%)	12(24%)
N1-N2 N(%)	15(30%)
N2A-N2C N(%)	18(72%)
N3-N3B N(%)	0(0%)
Unknown N(%)	5(10%)
**Tobacco Use**	
Yes N(%)	23(46%)
No N(%)	6(12%)
Former N(%)	14(28%)
Unknown N(%)	7(14%)
**ETOH use**	
Never N(%)	0(0%)
Yes N(%)	27(54%)
No N(%)	8(16%)
Occasional N(%)	2(4%)
Former N(%)	3(6%)
Unknown N(%)	10(20%)

Supplementary Table 3: Clinical characteristics of retrospective patient cohort for IHC and TLS analysis (Cohort 3)

**Panel 1**				**Panel 3**			
**VENDOR**	**CAT#**	**ANTIBODY/DYE**	**CLONE**	**VENDOR**	**CAT#**	**ANTIBODY/DYE**	**CLONE**
BD bioscience	741136	BUV496 Mouse Anti-Human CD7	(M-T701)	Biolegend	302262	Brilliant Violet 750™ anti-human CD19 Antibody	(HIB19)
BD bioscience	748443	BUV563 Mouse Anti-Human CD268 (BAFF Receptor)	(11C1)	ThermoFisher	62-0209-42	CD20 Monoclonal Antibody Super Bright 436, eBioscience™	(2H7)
BD bioscience	751138	BUV615 Mouse Anti-Human CD38	(HIT2)	Biolegend	348224	Pacific Blue™ anti-human IgD Antibody	(IA6-2)
BD bioscience	751680	BUV661 Mouse Anti-Human CD27	(O323)	BD bioscience	566146	BD Horizon™ BV480 Mouse Anti-Human IgM	(G20-127)
BD bioscience	741858	BUV737 Mouse Anti-Human IgG	(G18-145)	Biolegend	302825	Brilliant Violet 570™ anti-human CD27 Antibody	(O323)
BD bioscience	742007	BUV805 Mouse Anti-Human CD19	(HIB19)	BD bioscience	740414	BV605 Mouse Anti-Human CD23	(M-L233)
Biolegend	366314	APC/Fire™ 750 anti-human CD18 Antibody	(CBR LFA-1/2)	Biolegend	305440	Brilliant Violet 711™ anti-human CD86 Antibody	(IT2.2)
Biolegend	362906	Alexa Fluor® 488 anti-human CD191 (CCR1) Antibody	(5F10B29)	Biolegend	306526	PE/Dazzle™ 594 anti-human CD184 (CXCR4) Antibody	(12G5)
BD bioscience	742397	BD OptiBuild™ BV421 Mouse Anti-Human CD37	(M-B371)	ThermoFisher	46-9753-41	PerCP/EF710 anti-human SEMA4A	(clone 5E3)
BD bioscience	746638	BV480 Mouse Anti-Human CD54 (ICAM-1)	(HA58)	Biolegend	340304	PE anti-human CD307e (FcRL5) Antibody	(509f6)
BD bioscience	743625	BD OptiBuild™ BV510 Mouse Anti-Human CD180	(G28-8)	Biolegend	340202	Purified anti-human CD307d (FcRL4) Antibody	(413D12)
BD bioscience	743796	BD OptiBuild™ BV605 Mouse Anti-Human CD72	(J4-117)	Biolegend	304010	PE/Cyanine5 anti-human CD45 Antibody	(HI30)
BD bioscience	563922	BD Horizon™ BV650 Mouse Anti-Human CD196 (CCR6)	(11A9)	Biolegend	342804	PerCP/Cyanine5.5 anti-human CD305 (LAIR1) Antibody	(NKTA255)
BD bioscience	747484	BD OptiBuild™ BV750 Mouse Anti-Human IgD	(IA6-2)	BD bioscience	565156	BD Horizon™ BB515 Mouse Anti-Human CD70	(Ki-24)
BD bioscience	740969	BD OptiBuild™ BV786 Mouse Anti-Human CD21	(B-ly4)	Biolegend	310932	Brilliant Violet 785™ anti-human CD69 Antibody	(FN50)
BD bioscience	551058	PE-Cy™5 Mouse Anti-Human CD83	(HB15e)	BD bioscience	740570	BV650 Mouse Anti-Human CD22	(HIB22)
BD bioscience	564071	BUV395 Mouse Anti-Ki-67	(B56)	Biolegend	307620	Alexa Fluor® 488 anti-human HLA-DR Antibody	(L243)
ThermoFisher	46-9753-41	PerCP/EF710 anti-human SEMA4A	(clone 5E3)	Biolegend	356518	Brilliant Violet 510™ anti-human CD138 (Syndecan-1) Antibody	(MI15)
Biolegend	305406	PE anti-human CD86 Antibody	(IT2.2)	BD bioscience	566670	Alexa Fluor® 647 Mouse Anti-Human VISTA	(MIH65)
BD bioscience	563582	PE-Cy™7 Mouse Anti-Bcl-6	(K112-91)	Biolegend	356608	PE/Cyanine7 anti-human CD38 Antibody	(HB-7)
Biolegend	302332	Brilliant Violet 570™ anti-human CD20 Antibody	(2H7)	**Panel 4**			
Biolegend	300330	Pacific Blue™ anti-human CD3 Antibody	(HIT3a)	BD biosciences	565972	BD Horizon™ BUV395 Mouse Anti-Human HLA-DR	(G46-6)
Biolegend	340306	APC anti-human CD307e (FcRL5)	(509f6)	BD biosciences	612757	BD Horizon™ BUV737 Mouse Anti-Human CD19	(SJ25C1)
Biolegend	334328	Alexa Fluor® 700 anti-human CD40	(5C3)	Biolegend	305432	Brilliant Violet 510™ anti-human CD86 Antibody	(IT2.2)
BD bioscience	752351	BD OptiBuild™ BUV615 Mouse Anti-Human CD38	(HB7)	Biolegend	302828	Brilliant Violet 650™ anti-human CD27 Antibody	(O323)
**Panel 2**				Biolegend	329722	Brilliant Violet 711™ anti-human CD274 (B7-H1, PD-L1) Antibod	(29E.2A3)
Biolegend	302262	Brilliant Violet 750™ anti-human CD19 Antibody	(HIB19)	Biolegend	310932	Brilliant Violet 785™ anti-human CD69 Antibody	(FN50)
Biolegend	302332	Brilliant Violet 570™ anti-human CD20 Antibody	(2H7)	Biolegend	354906	APC anti-human CD21 Antibody	(Bu32)
Biolegend	354920	APC/Fire™ 750 anti-human CD21 Antibody	(Bu32)	Biolegend	334328	Alexa Fluor® 700 anti-human CD40 Antibody	(5C3)
Biolegend	302828	Brilliant Violet 650™ anti-human CD27 Antibody	(O323)	Biolegend	356604	PE anti-human CD38 Antibody	(HB-7)
Biolegend	348224	Pacific Blue™ anti-human IgD Antibody	(IA6-2)	ThermoFisher	61-2799-42	CD279 (PD-1) Monoclonal Antibody PE-eFluor 610	(eBioJ105 (J105))
BD bioscience	566146	BD Horizon™ BV480 Mouse Anti-Human IgM	(G20-127)	Biolegend	356513	PE/Cyanine7 anti-human CD138 (Syndecan-1) Antibody	(MI15)
Biolegend	340304	PE anti-human CD307e (FcRL5) Antibody	(509f6)	Biolegend	302326	PerCP/Cyanine5.5 anti-human CD20 Antibody	(2H7)
Biolegend	340202	Purified anti-human CD307d (FcRL4) Antibody	(413D12)	Miltenyi	130-113-475	FITC anti-human IgA	(IS11-8E10)
Biolegend	328214	Brilliant Violet 421™ anti-human CD39 Antibody	(A1)	**Panel 5**			
BD bioscience	563198	BV510 Mouse Anti-Human CD73	(AD2)	BD biosciences	741823	BD OptiBuild™ BUV737 Mouse Anti-Human CD4	(RPA-T4)
Biolegend	644835	Brilliant Violet 785™ anti-T-bet Antibody	(4B10)	BD biosciences	563795	BD Horizon™ BUV395 Mouse Anti-Human CD8	(RPA-T8)
Biolegend	301614	APC anti-human CD11c Antibody	(clone 3.9)	Biolegend	644820	Brilliant Violet 711™ anti-T-bet Antibody	(4B10)
BD bioscience	565153	BB515 Mouse Anti-Human LAIR-1 (CD305)	(DX26)	BD biosciences	563582	BD Pharmingen™ PE-Cy™7 Mouse Anti-Bcl-6	(K112-91)
Biolegend	307621	Alexa Fluor® 647 anti-human HLA-DR Antibody	(L243)	Biolegend	356928	PE/Dazzle™ 594 anti-human CD185 (CXCR5) Antibody	(J252D4)
Biolegend	334328	Alexa Fluor® 700 anti-human CD40 Antibody	(5C3)	ThermoFisher	48-4776-42	FOXP3 Monoclonal Antibody eFluor 450, eBioscience™	(PCH101)
Biolegend	306526	PE/Dazzle™ 594 anti-human CD184 (CXCR4) Antibody	(12G5)	Biolegend	302828	Brilliant Violet 650™ anti-human CD27 Antibody	(O323)
Biolegend	305440	Brilliant Violet 711™ anti-human CD86 Antibody	(IT2.2)	**Panel 6**			
Biolegend	3556641	Brilliant Violet 605™ anti-human CD38 Antibody	(HB-7)	Biolegend	300514	APC anti-human CD4 Antibody	(RPA-T4)
Biolegend	356513	PE/Cyanine7 anti-human CD138 (Syndecan-1) Antibody	(MI15)	Biolegend	301012	PE/Cyanine7 anti-human CD8a Antibody	(RPA-T8)
BD bioscience	612856	BD Horizon™ BUV737 Mouse Anti-Human CD70	(Ki-24)	Biolegend	644810	PE anti-T-bet Antibody	(4B10)
BD bioscience	744103	BD OptiBuild™ BUV395 Mouse Anti-Human CD178	(NOK1)	ThermoFisher	48-4776-42	FOXP3 Monoclonal Antibody eFluor 450, eBioscience™	(PCH101)
Biolegend	356912	Alexa Fluor® 488 anti-human CD185 (CXCR5) Antibody	(J252D4)	Biolegend	356928	PE/Dazzle™ 594 anti-human CD185 (CXCR5) Antibody	(J252D4)

Supplementary Table 4: Flow cytometry antibody panels

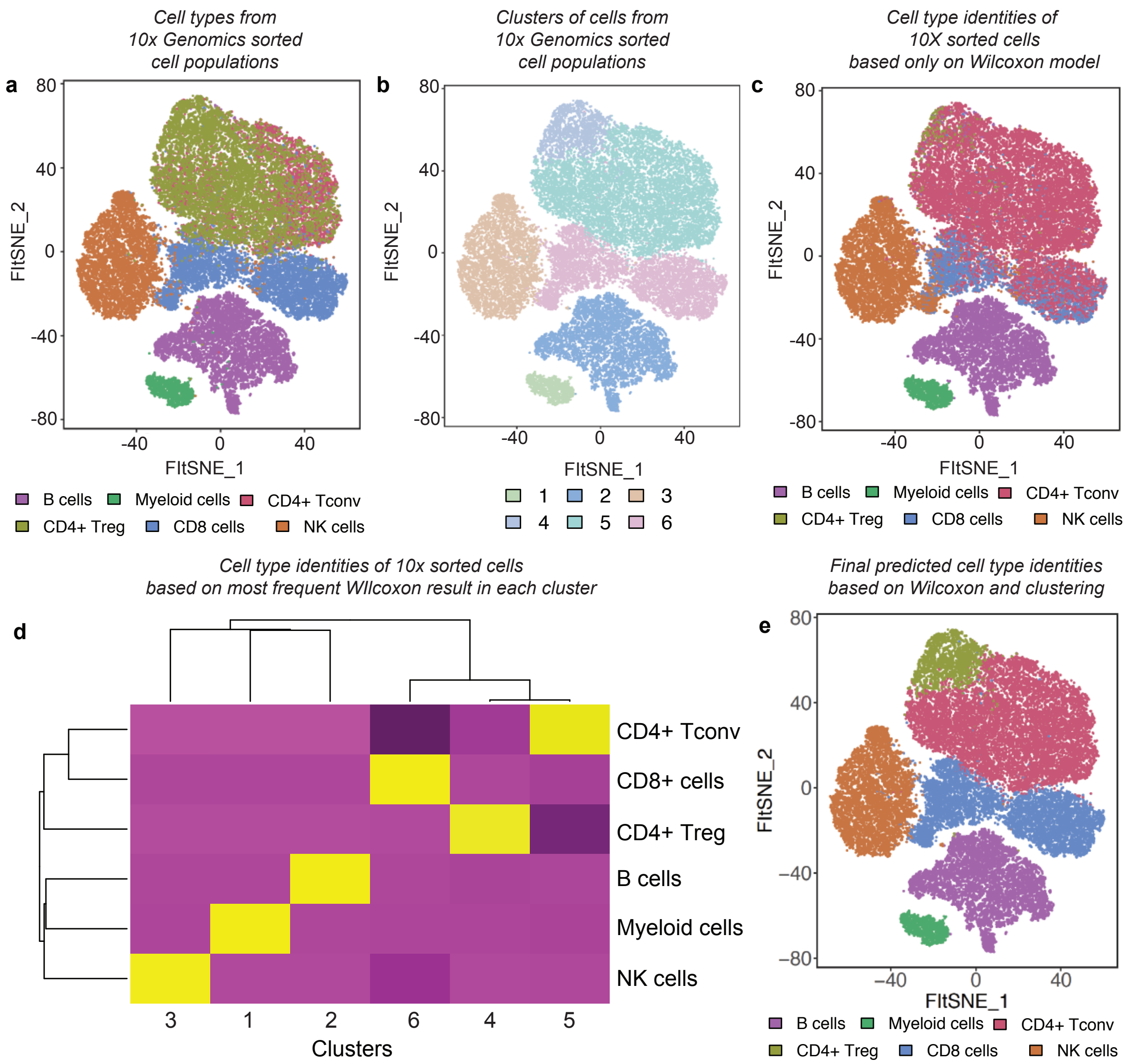

f

Metrics of cell type predictions from Wilcoxon and clustering model using sorted cells from 10X Genomics

	B cells	Myeloid cells	CD4+ Tconv	CD4+ Treg	CD8+ cells	NK cells
Sensitivity	1.000	0.987	0.976	0.928	0.990	0.985
Specificity	0.999	1.000	1.000	1.000	0.981	0.997
Pos Pred Value	0.997	0.996	1.000	1.000	0.926	0.987
Neg Pred Value	1.000	1.000	0.987	0.995	0.998	0.997
Prevalence	0.191	0.032	0.354	0.068	0.194	0.161
Detection Rate	0.191	0.031	0.346	0.063	0.192	0.158
Detection Prevalence	0.192	0.031	0.346	0.063	0.208	0.161
Balanced Accuracy	1.000	0.993	0.988	0.964	0.986	0.991

Supplementary Fig. 1: Validation of a combination Wilcoxon rank sum test and clustering based method for identification of cell types. Publicly available data from sorted populations of immune cells was combined, and a classification algorithm was employed to identify cells types. **a.** FItSNE plot of combined purified B cells, CD14+ monocytes, CD4+ helper T cells, CD4+ Treg, CD8+ T cells, and CD4+ regulatory T cells. **b.** Same FItSNE plot as (**a**), but showing clustering results. Clusters were strongly associated with cell types of purified populations. CD4+ Treg, despite overlapping with CD4+ Tconv as a purified population, were strongly associated with cluster 5, suggesting that the sorted population of CD4+ Treg were mixed with CD4+ Tconv. **c.** Raw results from testing of the Wilcoxon rank sum from known cell populations. Individual cells were scored for enrichment of markers associated with each purified cell population. Some clusters were readily identifiable as pure populations using just the Wilcoxon based enrichment, but mixtures of T cells were evident. **d.** Heatmap showing the association between inferred lineage type from the Wilcoxon rank sum scores and the clusters. Each cluster largely consisted of a major lineage when looking at the aggregate Wilcoxon rank sum test across clusters. **e.** Inferred cell types based on the association between Wilcoxon scores and clusters from (**d**). Cell type inference agreed strongly with results of clustering from (**b**). **f.** Confusion matrix comparing the inferred cell types to the ground truth. The sensitivity, specificity, and accuracy were between 0.93 and 1.0 for all lineages. Samples were derived from one blood donor for analysis.

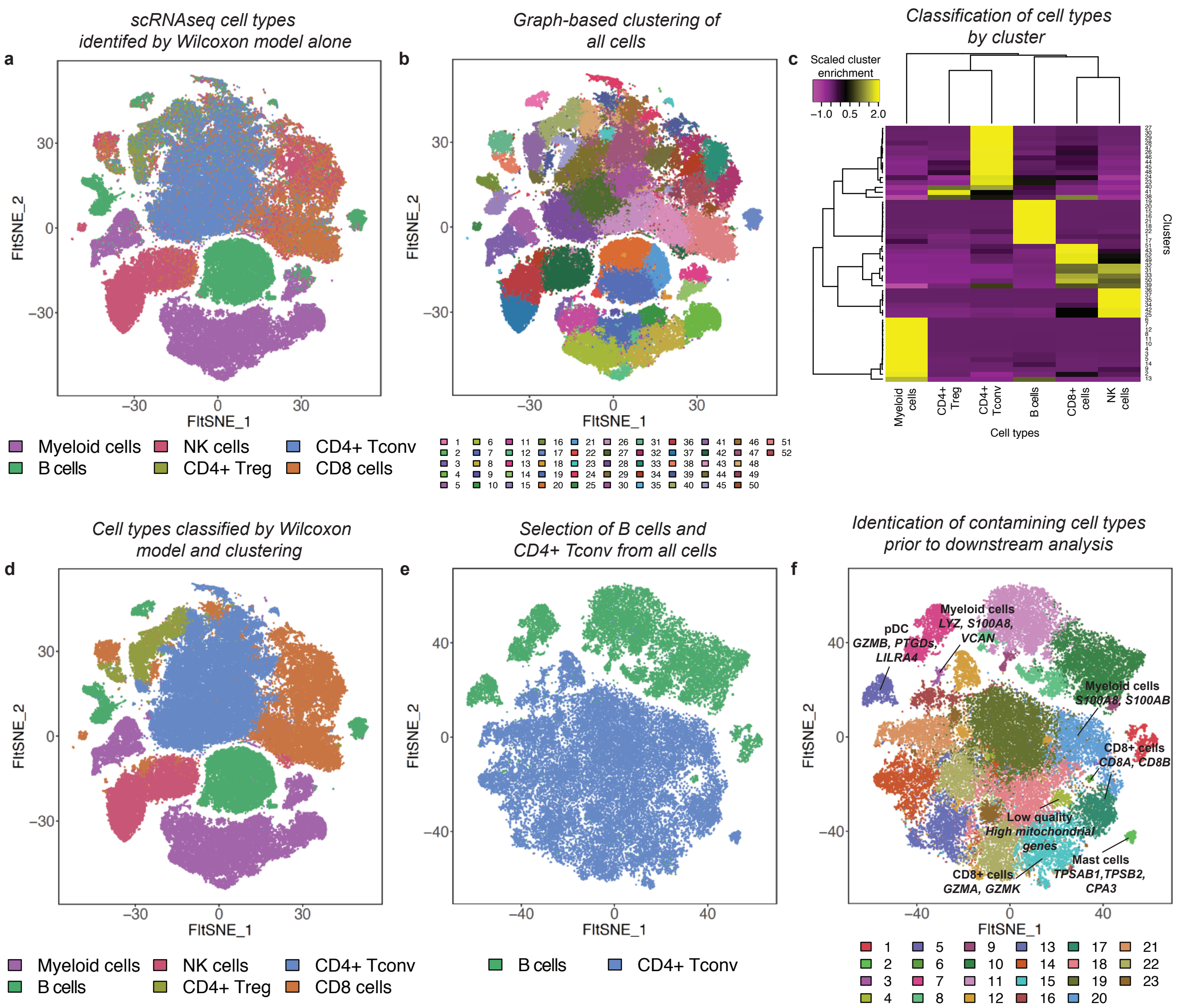

Supplementary Fig. 2: Identification of cell types from patients and controls using the combination Wilcoxon rank sum test and clustering based approach.

a. Raw results from the Wilcoxon scores derived for each individual cell. Certain populations are highly accurately inferred from this first step, while others exhibit mixtures of cell types. **b.** Results of Louvian clustering revealed a total of 52 clusters from all the cells in the dataset. **c.** Heatmap showing the relationship between cluster and inferred cell types from (**a**) and (**b**). **d.** Results of the combination of Wilcoxon scoring and cluster association from (**a-c**). Major

lineages are grouped together in FItSNE space, allowing the isolation of B cells and CD4+ Tconv for downstream analysis. **e.** B cells and CD4+ Tconv were bioinformatically isolated from D) and were projected in a new FItSNE space and colored by their inferred cell types. **f.** Identities of cell clusters were cross-checked by investigating differentially expressed genes across lineages. Contaminating lineages (i.e. those that are not B or CD4+ Tconv) and cell types that were not present in the training dataset (e.g. plasmacytoid dendritic cells [pDC], mast cells) were identified and removed, leaving only highly purified B and CD4+ Tconv for downstream analysis. Samples were derived from 6 healthy donors, 5 healthy tonsils, and 27 HNSCC patients.

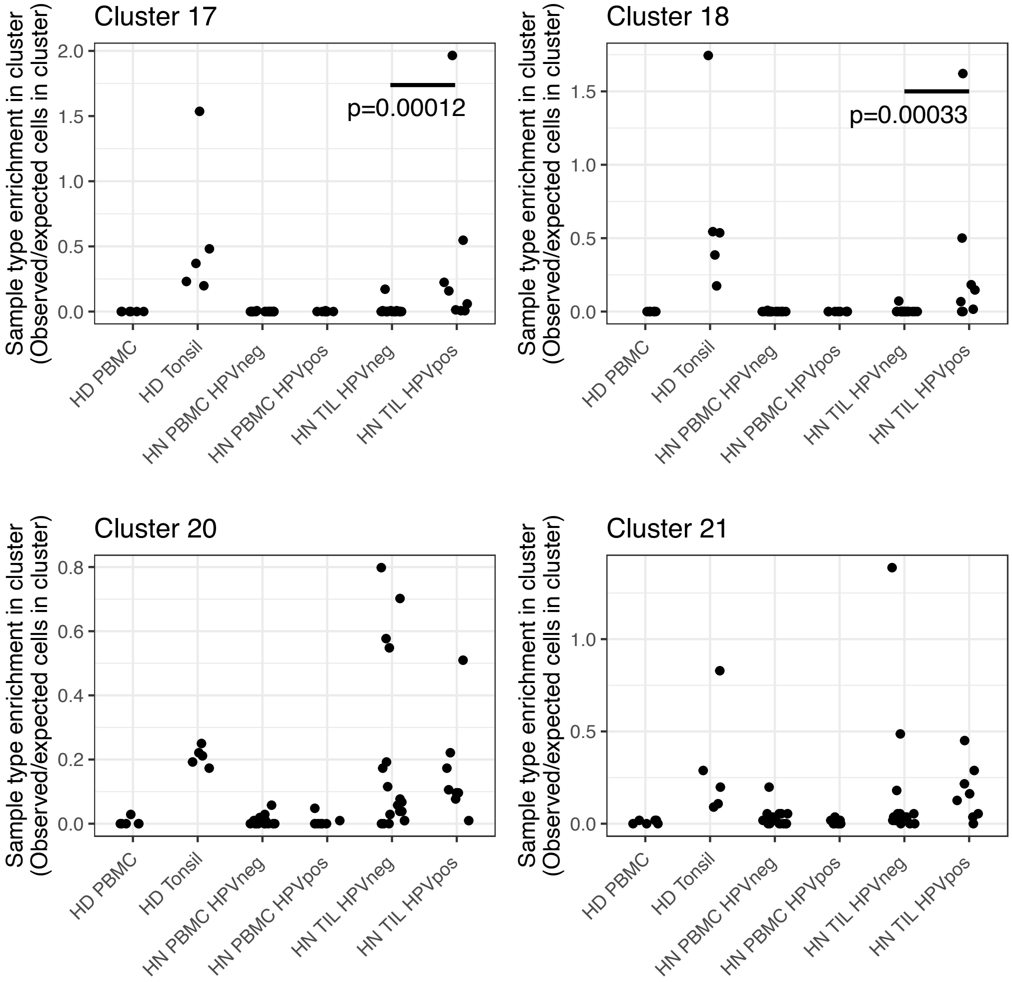

Supplementary Fig. 3: Statistical assessment of observed versus expected number of cells in germinal center and plasma cell clusters.

We first performed ANOVA tests to determine if there were differences between patient groups in a given cluster, and we found that clusters 17 (p=0.00033), 18 (p<0.0001) and 20 (p=0.0025) had at least one group that was different, while cluster 21 did not (p=0.067). Within clusters 17, 18 and 20 we tested whether there was any difference between TIL samples from HPV– versus HPV+ patients by Wilcoxon rank sum test and found that there were significantly higher frequencies of germinal center B cells in clusters 17 and 18. There was no different between HPV– versus HPV+ patients in for plasma cells in cluster 20. Source data are provided as a Source Data file. Samples were derived from 6 healthy blood donors, 5 healthy tonsils and 27

HNSCC patients.

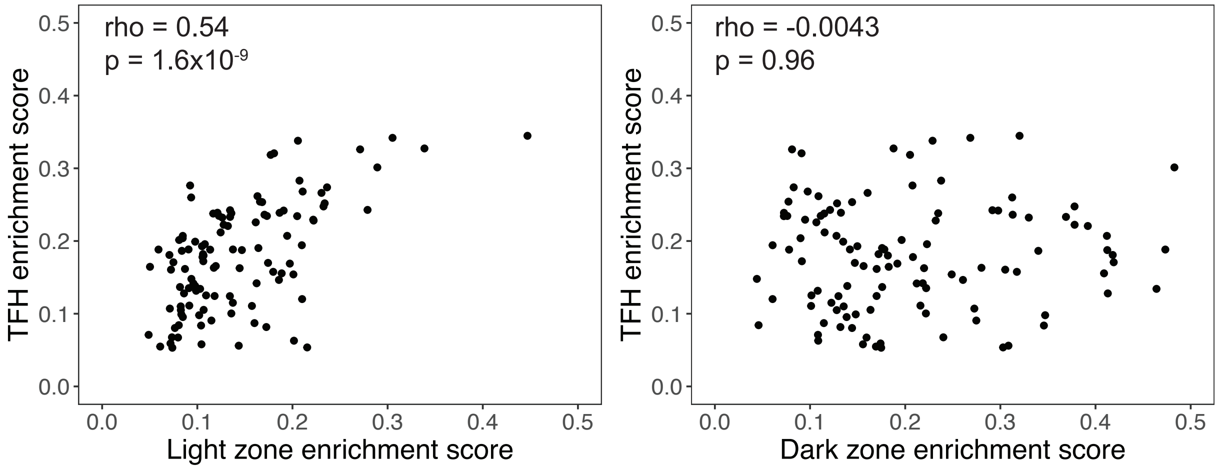

Supplementary Fig. 4. Relationship between germinal center cell states and TFH cells in samples from The Cancer Genome Atlas.

Light zone B cells were significantly correlated with TFH cells (left panel), but there was no relationship between dark zone B cells and TFH cells (right panel). Spearman's correlation was used, and p values are two-tailed. Source data are provided as a Source Data file. Samples were derived from 111 patients from the TCGA.

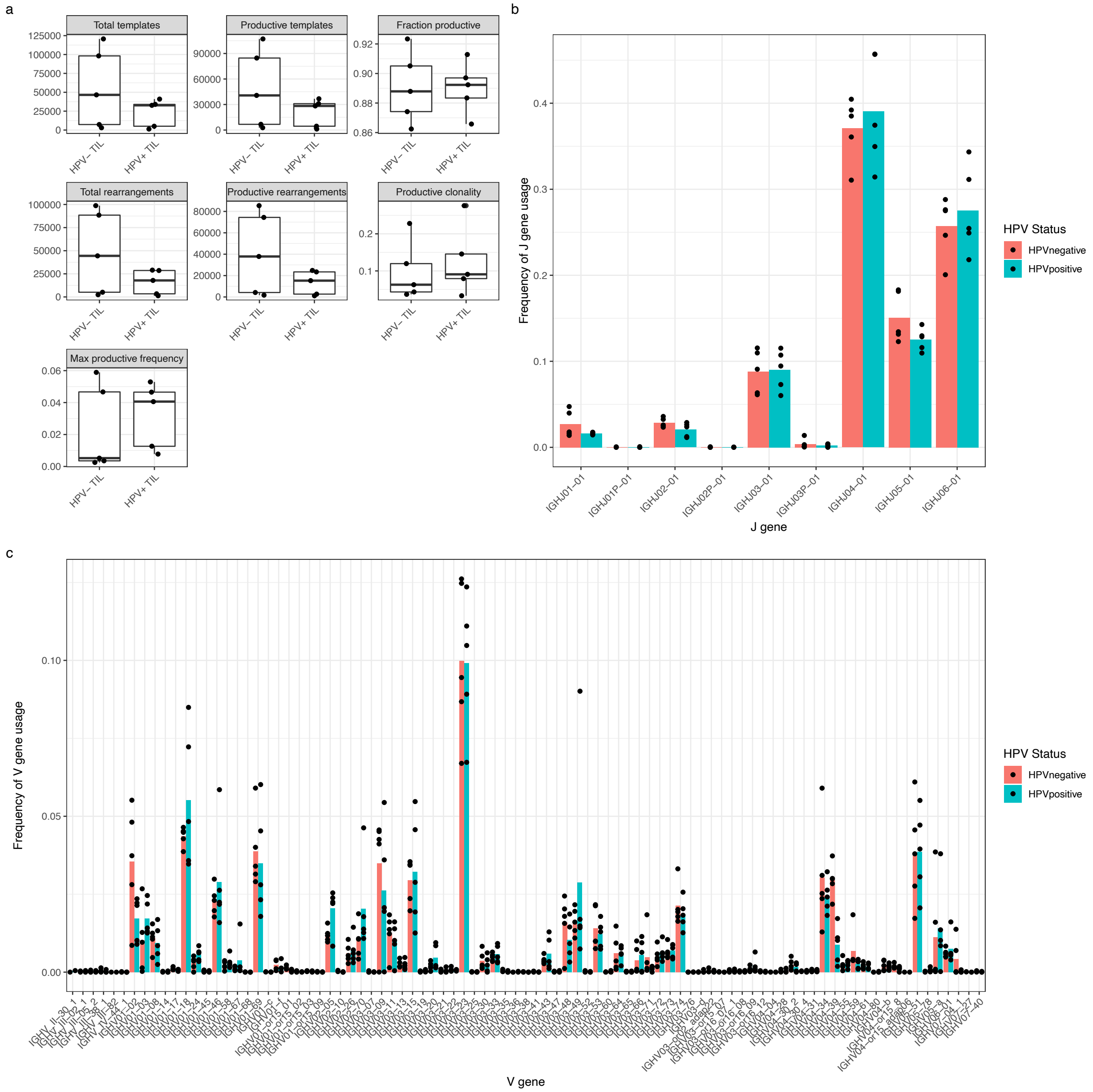

Supplementary Fig. 5: Adaptive BCR sequencing reveals no difference in clonality or other metrics between HPV– and HPV+ TIL.

a. There were no statistically significant differences by Wilcoxon rank sum test in BCR templates, rearrangements or clonality between HPV– and HPV+ TIL. BCR metrics were assessed from N=5 patients in each group. The line in the middle of the boxplot indicates the median, the top and bottom of the boxplot indicate the first and third quartiles, and the whiskers indicate 95% confidence intervals. **b.** There were no significant differences in J gene usage for

BCRs between HPV+ and HPV– TIL. J gene usage was assessed from N=5 patients in each group and compared by Wilcoxon rank sum test. **c.** There were no significant differences in V gene usage between BCRs from HPV– and HPV+ TIL. V gene usage was assessed from N=5 patients in each group and compared by Wilcoxon rank sum test. Source data are provided as a Source Data file.

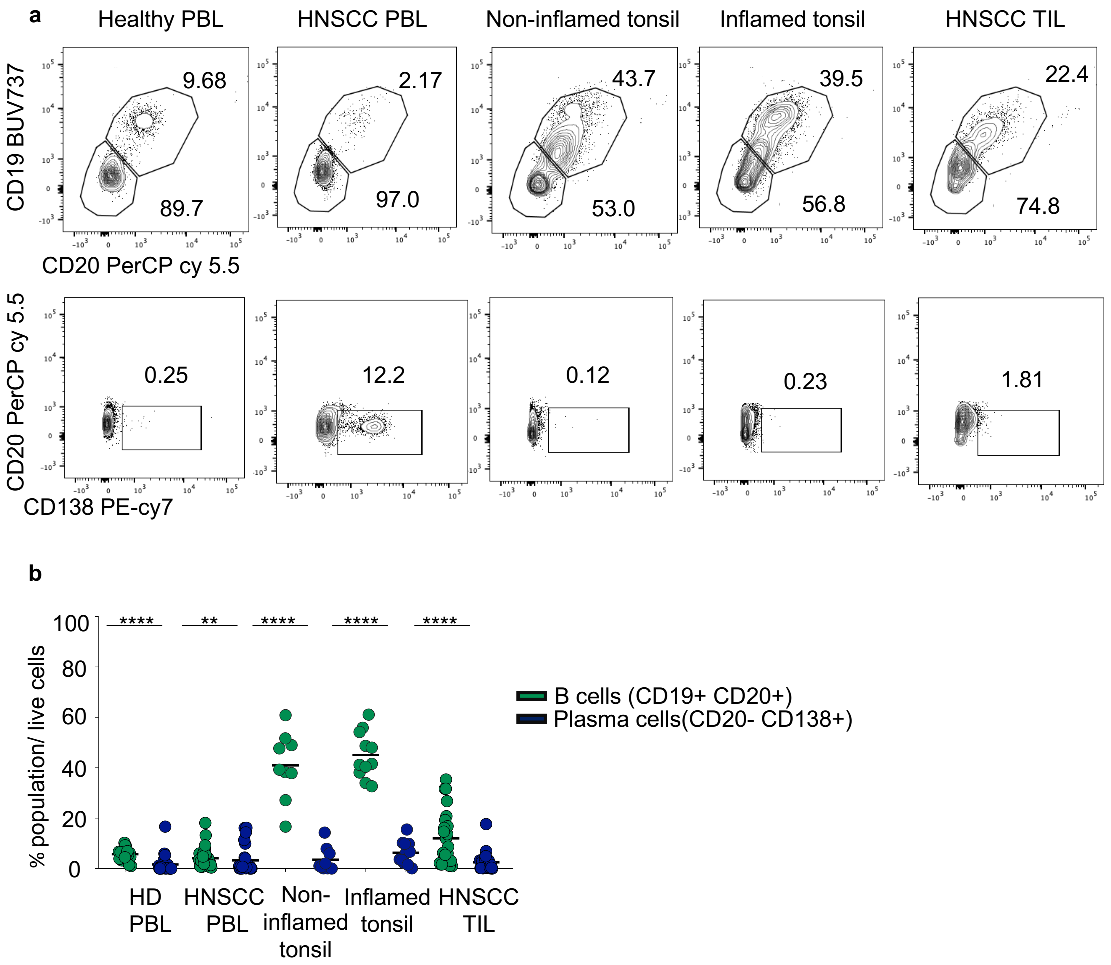

Supplementary Fig. 6: B cells are significantly increased compared to plasma cells in HNSCC patients.

a. Representative flow plots for quantification of B cell frequency compared to plasma cell frequency from a separate cohort of tonsils, healthy PBMCs, HNSCC TIL, and HNSCC PBMC.

b. Mean scatter plot showing the frequency of B cells compared to plasma cells in healthy PBL (n=22), non-inflamed tonsils (n=9), inflamed tonsil (n=11), HNSCC tumor (n=23), HNSCC PBMCs (n=30). Statistical analysis by Students Two-sided T-test (Mann-Whitney). ****P=<0.0001, **P=0.003. Source data are provided as a Source Data file.

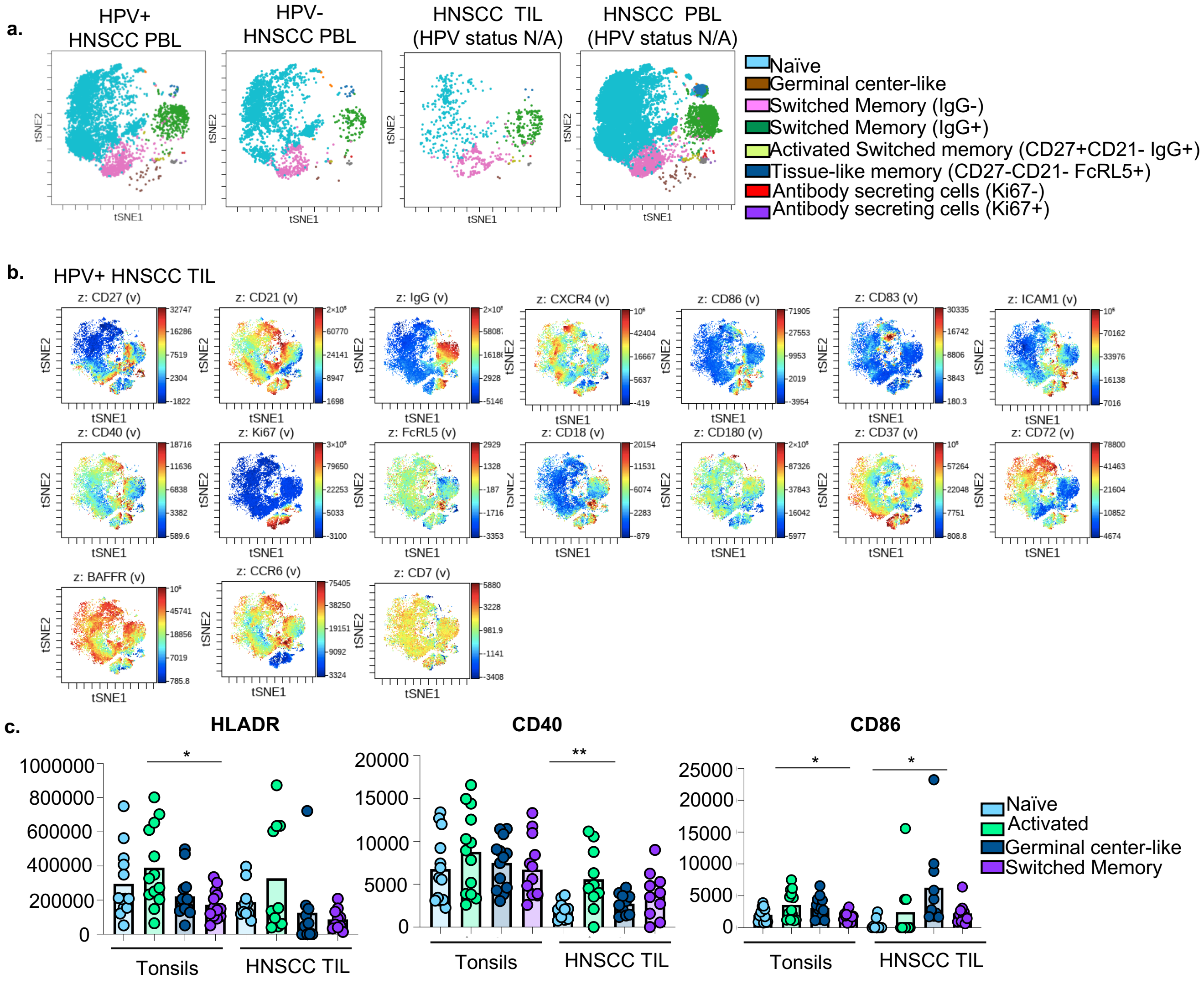

Supplementary Fig. 7: Additional high dimensional analysis of HNSCC cohort 2.

a. tSNE plots showing additional samples that were analyzed using Cytobank with patients from HNSCC cohort 2. Included were matched HPV+ (n=3) and HPV-PBMC (n=2). An additional set of HNSCC TIL (n=3) and PBL (n=14) were also included, however HPV status in these patients was not evaluated (HPV status N/A). **b.** Individual feature plots from HPV+ HNSCC patients demonstrating expression level of additional markers used to identify B cell subpopulations. **c.** Bar plot showing mean fluorescent intensity of HLADR, CD86, CD40 on B cell subsets. HNSCC TIL n=10 Tonsils n=13 Statistical analysis by one-way ANOVA followed by Tukeys multiple comparison. *P=0.03, **P=0.002. Source data are provided as a Source Data file

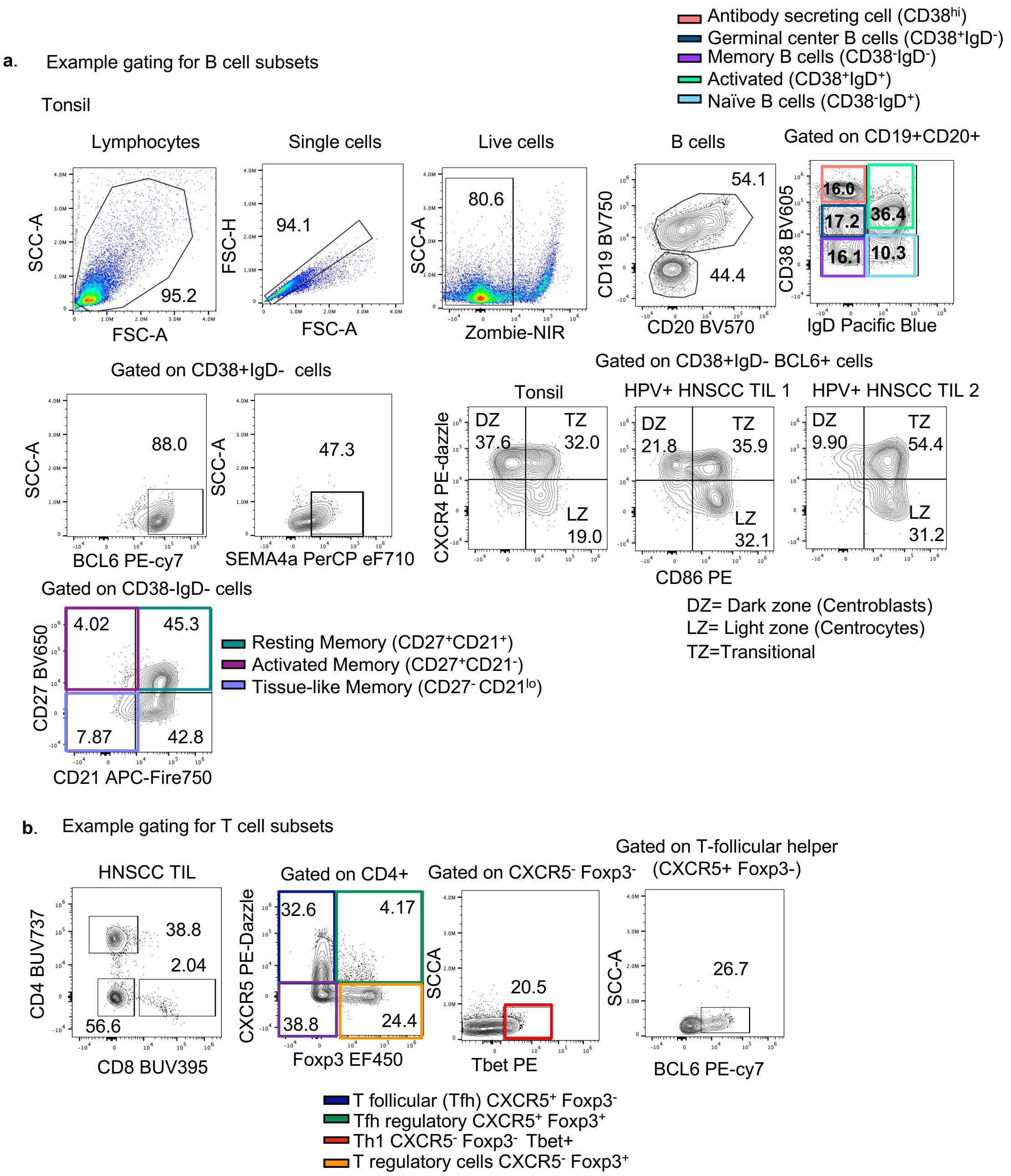

Supplementary Fig. 8: Flow cytometry gating strategy for B cell and T cell profiling.

a. Representative flow cytometry plots for analysis of samples stained with 25 parameter Cytek Aurora panel (**Fig. 2 and 4**). **b**. Representative flow cytometry plots for analysis of samples stained with T cell panel (**Fig. 2c**).

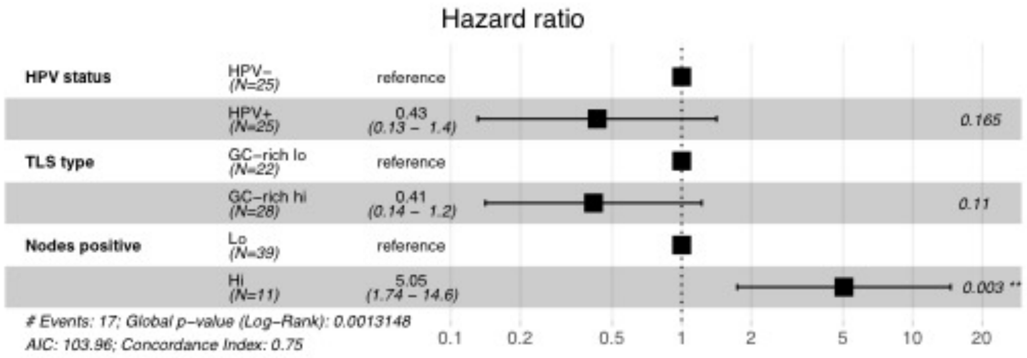

Supplementary Fig. 9: Multivariate survival analysis for TLS, HPV status, and disease burden.

Multivariate survival analysis based on HPV status, TLS type (i.e. either high TLS with GC or low TLS with GC) and disease burden as measured by the number of positive nodes. This multivariate analysis was significant with a log-rank p value of 0.0013; however, given the relatively small size of this dataset, neither the HPV status or TLS type were statistically significant after correcting for disease burden. Both HPV+ disease and high TLS with GC trended towards better outcomes. Source data are provided as a Source Data file. Samples were derived from 50 HNSCC patients.
