# Supplementary material for: B cell signatures and tertiary lymphoid structures contribute to outcome in head and neck squamous cell carcinoma
Source: Nat Commun. 2021 Jun 7;12:3349. doi: 10.1038/s41467-021-23355-x (PMC8184766; doi:10.1038/s41467-021-23355-x)
Supplement: Supplementary file 2 — Descriptions of Additional Supplementary Files [file 41467_2021_23355_MOESM2_ESM.pdf]

Descriptions of Additional Supplementary Files

Supplementary Data 1
Description: Differentially expressed genes across major canonical immune cell types.

Supplementary Data 2
Description: Differentially expressed genes across B cell and CD4+ Tconv clusters.

Supplementary Data 3
Description: Functional enrichment of protein-protein interactions occurring during the differentiation of naïve B cells to GC B cells.

Supplementary Data 4
Description: Raw BCR sequencing data from Adaptive
